# Adjuvant endocrine treatment strategies for non-metastatic breast cancer: a network meta-analysis

**DOI:** 10.1016/j.eclinm.2025.103116

**Published:** 2025-02-17

**Authors:** Andri Papakonstantinou, Guillermo Villacampa, Victor Navarro, Mafalda Oliveira, Antonios Valachis, Tomas Pascual, Alexios Matikas

**Affiliations:** a Oncology/Pathology Department, Karolinska Institutet, Stockholm, Sweden; b Breast Center, Karolinska Comprehensive Cancer Center, Stockholm, Sweden; c SOLTI Cancer Research Group, Barcelona, Spain; d Statistics Unit, Vall d'Hebron Institute of Oncology, Barcelona, Spain; e Medical Oncology Department, Vall d'Hebron University Hospital, and Breast Cancer Group, Vall D'Hebron Institute of Oncology, Barcelona, Spain; f Department of Oncology, Faculty of Medicine and Health, Örebro University, Örebro, Sweden; g Translational Genomics and Targeted Therapies in Solid Tumors, August Pi i Sunyer Biomedical Research Institute (IDIBAPS), Barcelona, Spain; h Medical Oncology Department, Hospital Clinic of Barcelona, Barcelona, Spain; i Faculty of Medicine and Health Sciences, University of Barcelona, Barcelona, Spain

**Keywords:** Aromatase inhibitor, Breast cancer, Endocrine treatment, Estrogen receptor, Tamoxifen

## Abstract

**Background:**

Multiple trials have evaluated escalation strategies of endocrine therapy for early breast cancer, including ovarian function suppression (OFS) and aromatase inhibitors (AI) in premenopausal patients and extended endocrine therapy. However, several aspects remain controversial due to the heterogeneity of study designs and lack of statistical power in relevant subgroups. We aimed to investigate the optimal endocrine therapy strategy.

**Methods:**

A systematic literature search was performed and last updated in August 2024 to identify randomized controlled trials (RCT) evaluating endocrine treatment strategies for hormone receptor positive breast cancer. A network meta-analysis with a frequentist framework using random-effects model was used to pool direct and indirect evidence. In addition, an extracted individual patient data meta-analysis was conducted to estimate the absolute differences between treatments. Study endpoints were disease-free survival (DFS), overall survival (OS), and safety. PROSPERO: CRD42023447979.

**Findings:**

A total of 37 RCT that had enrolled 107,684 patients were included in the study. During the first five years, OFS + AI was the most effective strategy in premenopausal women, while AI or switch strategy showed the better efficacy results in postmenopausal ones. Following five years of tamoxifen, continuation with five additional years of AI was associated with improved 8-year DFS (85.8%) compared to no extended therapy (78.1%) or five additional years of tamoxifen (81.0%). Following five years of AI or switch strategy, extended treatment with AI improved DFS (Hazard Ratio = 0.81, 95% Confidence Interval 0.73–0.90).

**Interpretation:**

This study provides information regarding the optimal endocrine treatment strategies for patients with resected hormone receptor positive early breast cancer.

**Funding:**

None.

**Research in context**

**Evidence before this study**

Endocrine treatment is broadly recommended to patients with resected estrogen receptor positive breast cancer, with new strategies (aromatase inhibitors, ovarian function suppression, molecularly targeted agents, and extended endocrine therapy) adding incremental benefits to patient survival. Heterogeneity in study design, control arms and endpoint definition, and lack of statistical power preclude uniform recommendations. To resolve this issue, we performed a systematic literature search of four databases (Medline, Embase, Cochrane Library, and Web of Science Core Collection) which was last updated in August 2024, a trial-level network meta-analysis with a frequentist framework, an analysis of reconstructed individual data from 107,684 patients treated within 37 randomized trials and a pooled analysis of safety data.

**Added value of this study**

This study provides evidence on the optimal management of premenopausal patients at lower risk of recurrence and the approach to extended endocrine therapy depending on administered treatment during the first five postoperative years. In addition, we quantify the expected absolute benefit in terms of improvement in disease free survival and number of patients needed to treat to avoid one relapse and present pooled safety data, enabling thus shared decision-making between patients and treating physicians.

**Implications of all the available evidence**

While endocrine treatment is one of the pillars of systemic postoperative breast cancer treatment, its efficacy needs to be balanced against its potential to cause side effects and negatively impact quality of life. The available evidence demonstrates that an optimal treatment strategy reduces the risk of recurrence by two thirds.

## Introduction

The recognition of the predictive value of estrogen receptor (ER) expression for benefit from adjuvant endocrine therapy (ET) following breast cancer resection led to the widespread recommendation of ET to all patients with ER-positive tumors. This recommendation was based on two observations: the associated reduction in breast cancer mortality derived from five years of adjuvant tamoxifen (30% relative and 9.2% absolute risk reduction at fifteen years of follow-up^1^); and the fact that even patients with very small tumors derive clinically meaningful overall survival (OS) benefit.^2^ Since the Early Breast Cancer Trialists' Collaborative Group (EBCTCG) meta-analysis on the relevance of ER expression, the results of several randomized controlled trials (RCT) investigating newer agents (ovarian function suppression [OFS], aromatase inhibitors [AI] and molecularly targeted agents) have matured and have been summarized in further meta-analyses, showing continued, albeit in some cases borderline, improvements over tamoxifen.^3^^,^^4^

Furthermore, an individual patient data meta-analysis and a nationwide population-based study have clearly demonstrated the long natural history of ER-positive breast cancer.^5^^,^^6^ Multiple RCT have thus investigated the value of extending ET for durations that range from a total of seven years to indefinite treatment. Substantial differences in their design, agents used, marginal or no OS benefit in most trials and low power of individual RCT to detect specific subgroups benefiting the most, make it difficult to recommend extended treatment to all patients, or even offer specific guidance. As such, the decision to extend treatment is based purely on prognostic information (for example, with the use of the Clinical Treatment Score 5 algorithm^7^^,^^8^) and not on the benefit patients derive from treatment.

Network meta-analyses permit comparisons of multiple treatments at the same time by combining direct and indirect evidence within a network of RCT.^9^ In order to provide answers to these questions, previously unresolved due to either lack of statistical power or lack of RCT evaluating direct comparisons between specific strategies, we performed a trial-level network meta-analysis and an analysis of extracted individual patient data to investigate how different ET strategies impact the prognosis of patients with ER-positive early breast cancer. Our aims were to evaluate the optimal regimens for different patient subgroups during the first five years of treatment, to identify subpopulations most likely to benefit from extended endocrine therapy, and quantify the absolute benefit derived from different treatment strategies including targeted therapies for high-risk patients.

## Methods

We performed a systematic review of the literature to identify RCT that investigate ET strategies for early breast cancer, a network meta-analysis and a meta-analysis of extracted individual patient data. The article selection and meta-analysis were conducted and reported according to the Preferred Reporting Items for Systematic Reviews and Meta-Analyses (PRISMA) guidelines and the PRISMA extension for network meta-analysis.^10^ The network meta-analysis is registered in the International Prospective Register for Systematic Reviews (PROSPERO), with registration number CRD42023447979.

### Search strategy and study selection

The literature search included four databases: Medline (Ovid), Embase, Cochrane Library (Wiley), and Web of Science Core Collection (Clarivate) and was performed by librarians at Karolinska Institute and last updated in August 2024. Following the final search, the proceedings of the European Society for Medical Oncology annual meeting 2024 were searched manually and the results of one trial (NATALEE^11^) were updated. The search strategy was developed in Medline (Ovid) in collaboration with librarians at the Karolinska Institutet University Library. For each search concept Medical Subject Headings (MeSH-terms) and free text terms were identified. The search was then translated, in part using Polyglot Search Translator,^12^ into the other databases. No language restriction was applied and databases were searched from inception. Filters for randomized controlled trials were used in Medline, Embase, and Web of Science.^13^, ^14^, ^15^ The strategies were peer reviewed by another librarian prior to execution. De-duplication was done using the method described by Bramer et al.^16^ One final, extra step was added to compare digital object identifiers (DOIs). A snow-ball search was applied to check references and citations of eligible studies from the database searches using EndNote®. The complete search strategy for each database is available in Supplementary Data.

Eligible studies were RCT comparing different ET strategies. Studies comparing same class ET strategies (e.g., two AI strategies) were excluded. Peer reviewed conference abstracts with no available full publications were accepted if all relevant information was included. When several publications were available from the same trial, relevant data were extracted from different publications to pursue maximal coverage and the latest publication concerning each relevant endpoint was selected for the analyses, but the trial was only counted once. The updated Cochrane Risk of Bias assessment of randomized trials tool (RoB2) was utilized to evaluate risk of bias from the published manuscripts, performed by two authors (AP, AM).^17^

### Data extraction and outcomes

Two authors (AP and AM) performed data extraction from the selected trials and relevant publications to a predefined form. Extracted data included first author and trial name, year of publication, enrolled patients in total and per treatment arm, intervention and control treatment, median follow-up, survival outcomes and adverse events. Any inconsistencies were discussed and resolved. The primary outcome was disease-free survival, which was the primary endpoint in most trials (Table 1). Since the definitions of the primary endpoint across studies were relatively consistent in terms of local/distant relapse and death though not standardized to include the same events (Supplementary Table S1), the reported results were pooled in the meta-analysis. Secondary outcomes were overall survival (OS) and safety, with special focus on osteoporosis, fractures, hot flushes, deep-venous thrombosis and cardiovascular side effects, as well as discontinuation rates.

**Table 1:** Description of the studies included in the network meta-analysis, presented per treatment group.

First author, publication year	Study name	Identifier	Years of enrollment	Comparison	Sample size	Median follow-up (months)	Premenopausal	Node positive	Received chemotherapy	Primary endpoint	Region
**Strategies during first five postoperative years (9 trials, n = 33,614)**											
Kaufmann, 2007	ARNO-95	NCT00287534	1996–2002	5 years TAM vs switch	979	30	0%	26%	0%	DFS	E
Cuzick, 2010	ATAC	ISRCTN18233230	1996–2000	5 AI vs 5 years Tam	5216	120	0%	34%	20%	DFS	A, E, NAm, O, SA
Regan, 2011	BIG 1-98	NCT00004205	1998–2003	5 years TAM vs 5 years AI vs switch	8010	97	0%	39%	49%	DFS	A, Af, E, NAm, O, SA
Dubsky, 2012	ABCSG-8	NCT00291759	1996–2004	5 years TAM vs switch	3714	60	0%	25%	0%	RFS	E
Boccardo, 2013	ITA	NA	1998–2002	5 years TAM vs switch	448	128	0%	100%	66%	DFS	E
Aihara, 2014	N-SAS BC03	C000000056	2002–2005	5 years TAM vs switch	706	98.5	0%	40%	53%	DFS	A
Morden, 2017	IES	ISRCTN11883920	1998–2003	5 years TAM vs switch	4724	120	0%	44%	32%	DFS	A, E, NAm, O, SA
Derks, 2017	TEAM	NCT00032136	2001–2006	5 years AI vs switch	6120	116	0%	57%	63%	DFS	E
De Placido, 2018	FATA-GIM3	NCT00541086	2007–2012	Compare 5 years AIs and then with switch	3697	60	0%	36%	38%	DFS	E
**Strategies incorporating ovarian function suppression (9 trials, n = 15,910)**^a^											
ABCTCG, 2007	ABC OAS	ISRCTN31514446	1993–2000	OFS + Tam vs Tam	2144	59	100%	65%	20%	OS	E
Hackshaw, 2009	ZIPP	NA	1987–1999	OFS + Tam vs Tam	1761	144	100%	39%	NA	EFS	E
Tevaarwerk, 2014	E-3193/INT-0142	NA	1994–1997	OFS + Tam vs Tam	345	119	100%	0%	0%	DFS	NAm
Gnant, 2014	ABCSG-12	NCT00295646	1999–2006	OFS + AI vs OFS + Tam	1803	94	100%	31%	5%	DFS	E
Yang, ASCO 2016	NA	NCT00827307	2008–2009	OFS + Tam vs Tam	110	72	100%		NR	Estradiol, breast density, lipids	A
Perrone, 2019	HOBOE	NCT00412022	2004–2015	OFS + AI vs OFS + Tam	710	64	100%	45%	63%	DFS	E
Pagani, 2022	SOFT/TEXT	NCT00066690/NCT00066703	2003–2011	OFS + AI vs OFS + Tam	4690	156	100%	42%	46%	DFRI	A, Af, E, O, NAm, SA
Francis, 2022	SOFT	NCT00066690	2003–2011	OFS + AI vs OFS-Tam vs Tam	3047	144	100%	35%	53%	DFS	A, Af, E, NAm, O, SA
Baek, 2023	ASTRRA	NCT00912548	2009–2014	OFS + Tam vs Tam	1298	106	100%	54%	NR	DFS	A
**Extended endocrine therapy (13 trials, n = 37,195)**											
Fisher, 2001	NSABP-B14	NA	1987–1994	Addition of indefinite TAM to 5 years TAM	1152	84	26%	0%	NR	DFS	NAm
Stewart, 2001	Scottish	NA	1985–1989	Addition of indefinite TAM to 5 years TAM	342	103	NR	NR	NR	Relapse	E
Jakesz, 2007	ABCSG 6a	NCT00300508	NR	Additional 3 years AI to 5 years TAM	856	62	0%	67%	NR	RFS	E
Mamounas, 2008	NSABP B-33	NCT00016432	2001–2003	Additional 5 years AI to 5 years TAM	1598	30	0%	48%	55%	DFS	NAm
Ingle, 2008	MA.17	NCT00003140	1998–2002	Additional 5 years AI to 5 years TAM	5187	64	0%	46%	45%	DFS	E, NAm
Davies, 2013	ATLAS	ISRCTN19652633	1996–2005	Additional 5 years TAM to 5 years TAM	6846	91	10%	46%	NR	RFS	A, Af, E, NAm, O, SA
Gray, 2013	aTTom	ISRCTN17222211	1991–2005	Additional 5 years TAM to 5 years TAM	6953	NR	NR	NR	NR	DFI	A, Af, E, NAm, O, SA
Blok, 2018	IDEAL	2006-003958-16	2007–2011	Additional 5 vs 2.5 years to 5 years TAM/AI	1821	79	0%	75%	68%	DFS	E
Del Mastro, 2021	GIM4	NCT01064635	2005–2010	Additional 5 vs 2–3 years AI to 2–3 years TAM	2056	140	0%	44%	55%	DFS	E
Gnant, 2021	ABCSG-16	NCT00295620	2004–2010	Additional 5 vs 2 years AI to 5 years TAM/AI	3208	118	0%	33%	29%	DFS	E
Mamounas, 2023	NSABP B-42	NCT00382070	2006–2010	Additional 5 years AI to 5 years AI or TAM/AI	3923	124	0%	42%	NR	DFS	E, NAm
Iwase, 2023	AERAS	UMIN000000818	2007–2012	Additional 5 years AI to 5 years AI or TAM/AI	1593	59	0%	35%	60%	DFS	A
Tjan-Heijnen, 2023	DATA	NCT00301457	2006–2009	Additional 6 vs 3 years AI to 2–3 years TAM	1660	121	0%	66%	68%	DFS	E
**Addition of targeted agents (6 trials, n = 20,965)**											
Mayer, 2021	PALLAS	NCT02513394	2015–2018	ET ± palbociclib	5760	23.7	53%	87%	82%	iDFS	A, E, NAm, O, SA
Loibl, 2021	Penelope-B	NCT01864746	2014–2017	ET ± palbociclib	1250	42.8	49%	94%	100%	iDFS	A, E, NAm, O
Bachelot, 2022	UNIRAD	NCT01805271	2013–2020	ET ± everolimus	1278	35.7	32%	100%	100%	DFS	E
Chavez, 2024	SWOGS1207	NCT01674140	2013–2019	ET ± everolimus	1939	55.2	32%	91%	100%	iDFS	NAm
Hortobagyi, 2023	NATALEE	NCT03701334	2019–2021	ET ± ribociclib	5101	33.3	44%	60%	88%	iDFS	A, E, NAm, O, SA
Fasching, 2024	monarchE	NCT03155997	2017–2019	ET ± abemaciclib	5637	54	43%	99%	95%	iDFS	A, E, NAm, O, SA

Abbreviations: A: Asia; AEs: Adverse Events; Af: Africa; AI: Aromatase Inhibitor; DFS: Disease-Free Survival; E: Europe; EFS: Event-Free Survival; ET: endocrine therapy; iDFS: invasive Disease-Free Survival; Nam: North America; NA: Not Available; NR: Not Reported; O: Oceania; OFS: Ovarian Function Suppression; RFS: Relapse-Free Survival; SA: South America; TAM: Tamoxifen.

a SOFT listed twice, as individual trial and as part of the SOFT/TEXT pooled analysis.

### Statistics

Firstly, a trial-level network meta-analysis with a frequentist framework was performed to simultaneously compare all treatment strategies. The network meta-analysis incorporates i) direct estimations (traditional pairwise meta-analysis) and ii) indirect estimations, estimations of treatments that have been never compared head-to-head but have been evaluated in separate trials in comparison with a common control arm.^18^^,^^19^ To summarize efficacy endpoints, hazard ratios (HR) with unadjusted 95% confidence intervals (CI) were calculated for the DFS and OS analysis, with HR less than 1 indicating a risk reduction in the treatment defined as experimental arm. For the safety analysis, percentages and odds ratios (OR) with 95% CI were calculated, with OR greater than 1 indicating higher odds of adverse events in patients receiving the experimental arm. Random-effects models were fitted for all the analyses regardless of the amount of heterogeneity of the model (estimated by means of the $I^{2}$ statistic), assuming that there is not one true intervention effect but a distribution of true intervention effect. Fixed-effects models were also estimated as a sensitivity analysis but are not reported in this manuscript because the results were highly similar between the two models. To evaluate the transitivity assumption, we examined the network consistency using the net-splitting method to compare the direct and indirect evidence in all comparisons.^20^

Secondly, an extracted individual patient data meta-analysis was performed. Initially, we identified the Kaplan–Meier curves from all included studies that reported survival outcomes. The Kaplan–Meier curve with the longest follow-up duration was selected for each study. Data from both manuscripts or scientific conferences were included. We used the software DigitizeIt (http://www.digitizeit.de/) to extract data values from the Kaplan–Meier curves. This procedure allowed us to obtain the data coordinates and estimate the survival probabilities at each time point. A web-based Shiny app (https://www.trialdesign.org/one-page-shell.html#IPDfromKM) was utilized for reconstructing the individual patient-level data. The survival probabilities obtained in the second step were combined with i) the total number of patients included in each arm, ii) the pre-defined risk times (e.g., 0, 1, 2 years, etc.), iii) the number of patients at risk at each time point, and iv) the total number of events (if reported). The estimated individual-patient level data obtained in third step were used to generate Kaplan–Meier curves for each study. To evaluate the quality of the reconstruction, we compared the generated Kaplan–Meier curves with those reported in the original publications. The following items were evaluated: hazard ratios, shape of the Kaplan–Meier curves, survival rates at the time points where the original publication reported results (e.g., 5, 8 years, etc.), number at risk tables. If discrepancies were observed, we started from step 2 to initiate the reconstruction process. Only marginal differences were considered acceptable. Using the reconstructed data from each study, a pooled analysis was performed by combining information from patients receiving the same treatment strategy. Stratified Cox models were used to estimate HR using the extracted individual patient data, with the study name as a stratification factor. Additionally, to estimate the absolute differences between treatments over various follow-up years—not only relative differences in terms of HR but also percentage differences in survival rates—adjusted survival curves from Cox models were plotted using the conditional approach.^21^ This method minimizes potential prognostic differences between trials that may arise due to the different time periods in which the trials were conducted. The proportional hazards assumption was tested and visually inspected using Schoenfeld residuals, with no evidence of non-proportionality observed. We used the survival rates derived from the extracted individual patient data, to estimate the number needed to treat (NNT) to prevent one event.^22^

No imputation was performed and no p-values were reported to avoid multiple comparison and the potential overreporting of p-values. Publication bias was not assessed because traditional funnel plots are not well-suited for network meta-analysis. In such cases, asymmetry should not be interpreted as evidence of publication bias.^23^ All analyses were undertaken using R statistical software version 4.3.1 (packages meta 7.0-0, netmeta 2.9-0, ggplot2 3.5.1 and survival).

### Ethics

This study is a systematic review and meta-analysis of published clinical trials. As such, no approval from ethics committee was necessary. Regarding the ethics approval of each trial, the reader is referred to the individual publications.

### Role of funding source

There was no funding source for this study.

## Results

**Fig. 1:**
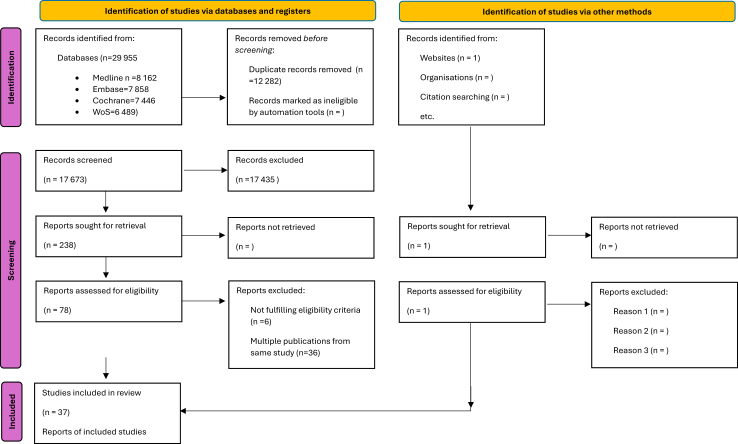
Preferred Reporting Items for Systematic Reviews and Meta-Analyses (PRISMA) flow diagram of search results.^10^ For more information, visit: http://www.prisma-statement.org/.

The literature search identified 17,673 records following deduplication (Fig. 1 and Supplementary Data). For the trial-level network meta-analysis, 6 studies were not included, as described in Supplementary Table S2. Thus, 37 studies that had enrolled 107,684 patients were included in the networks.^11^^,^^24^, ^25^, ^26^, ^27^, ^28^, ^29^, ^30^, ^31^, ^32^, ^33^, ^34^, ^35^, ^36^, ^37^, ^38^, ^39^, ^40^, ^41^, ^42^, ^43^, ^44^, ^45^, ^46^, ^47^, ^48^, ^49^, ^50^, ^51^, ^52^, ^53^, ^54^, ^55^, ^56^, ^57^, ^58^, ^59^, ^60^ A graphic description of the geographical distribution of the trials is presented in Supplementary Figure S1. The study characteristics are presented in Table 1. Evaluation of risk of bias according to the RoB2 tool revealed no concerns for the majority of the included trials and is presented in Supplementary Figure S2.

### Endocrine therapy during the first five postoperative years

#### Trial-level network meta-analysis

**Fig. 2:**
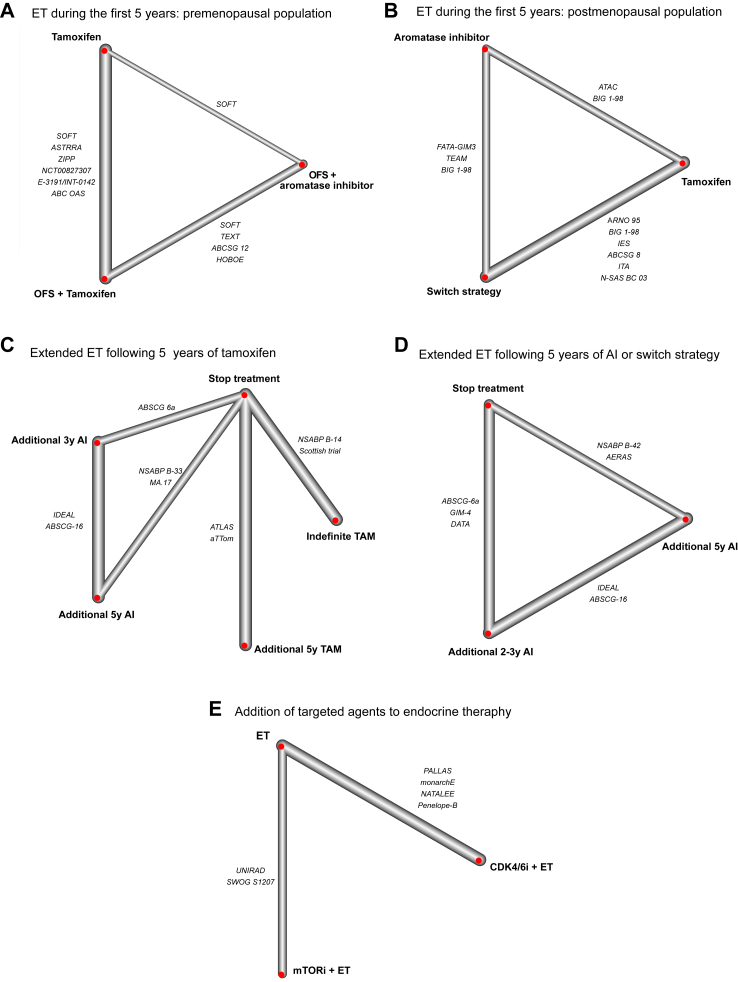
Network graph of adjuvant endocrine therapy strategies in early breast cancer. The lines connect treatments that have been compared head-to-head, and the width of lines is proportional to the number of studies that evaluated this pairwise comparison. A) Premenopausal patients, first five years. B) Postmenopausal patients, first five years. C) Extended endocrine therapy after five years of tamoxifen. D) Extended endocrine therapy after five years of aromatase inhibitors or switch strategy. E) Addition of molecularly targeted agents. Abbreviations: OFS: ovarian function suppression; AI: aromatase inhibitor; CDK4/6i: cyclin dependent kinases 4/6 inhibitor; mTORi: mammalian target of rapamycin inhibitor.

The network graph for the comparisons of ET during the first five postoperative years is shown in Fig. 2A for premenopausal (n = 13,856) and Fig. 2B for postmenopausal patients (n = 31,651). No evidence of inconsistency was observed in the networks (Supplementary Figure S3).

**Fig. 3:**
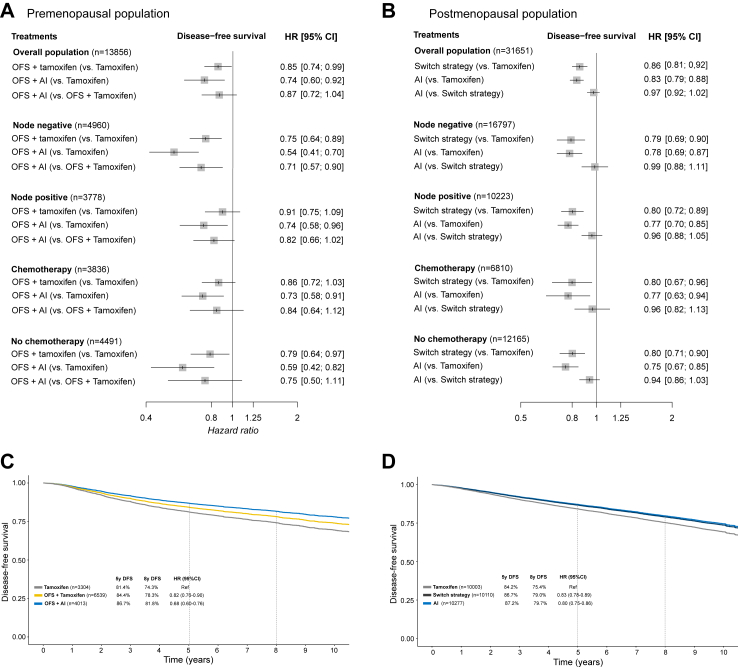
Comparison of treatment strategies during the first five postoperative years. Trial-level network meta-analysis for the disease-free survival endpoint in A) premenopausal and B) postmenopausal patients. Kaplan–Meier curves for disease-free survival, generated with extracted individual patient data, comparing C) three treatment strategies, Tamoxifen, OFS + Tamoxifen and OFS + AI in premenopausal women, and D) three treatment strategies, tamoxifen, aromatase inhibitors or switch strategy in postmenopausal women. Abbreviations: OFS: ovarian function suppression; AI: aromatase inhibitors; N: nodal status; DFS: disease-free survival; HR: hazard ratio; CI: confidence interval.

In the premenopausal population, three treatment strategies were compared: i) tamoxifen alone, ii) OFS + tamoxifen, and iii) OFS + AI. Compared with tamoxifen, both OFS + AI and OFS + tamoxifen were consistently associated with improved DFS in the total population and in subgroups defined according to nodal status and use of chemotherapy (Fig. 3). In lower risk groups such as patients with no nodal involvement or with no administered (neo)adjuvant chemotherapy, where tamoxifen monotherapy is commonly recommended, OFS + AI was associated with almost half the risk of recurrence compared to tamoxifen alone (node negative, HR = 0.54, 95% CI 0.41–0.70 and no chemotherapy, HR = 0.59, 95% CI 0.42–0.82). In the comparison OFS + AI vs OFS + tamoxifen, HR for DFS favored OFS + AI in all subgroups, although confidence intervals were wide in several comparisons. Moreover, both combination treatments were associated with improved OS compared to tamoxifen, although the effect was clearer in patients that had received chemotherapy. OFS + AI did not improve OS compared to OFS + tamoxifen (HR = 1.05, 95% CI 0.83–1.33) (Supplementary Figure S4).

In the postmenopausal population, three treatment strategies were evaluated: i) tamoxifen for five years, ii) AI for five years, and iii) switch during the first five years with either sequence (tamoxifen first followed by AI, or vice versa). AI only and switch strategy were consistently associated with improved DFS across all subgroups (Fig. 2) and OS in the total population (Supplementary Figure S4). In the AI versus switch comparison, there was no evidence of improvement in DFS or OS.

#### Extracted individual patient data meta-analysis

In the overall population of premenopausal patients, OFS + AI was associated with an absolute improvement in DFS at 8 years of 7.5% compared to tamoxifen (81.8% vs 74.3%, resulting in NNT to avoid one recurrence of 13), while a 3.5% absolute improvement over OFS + tamoxifen was observed (81.8% vs 78.3%). In the overall population of postmenopausal patients, 5 years with AI and switch strategy were associated with similar outcomes, both superior to 5 years of tamoxifen (8-year DFS 79.7% for AI, 79.0% for switch strategy and 75.4% for tamoxifen) (Fig. 3).

#### Safety

In premenopausal women, pooled rates of deep venous thrombosis (DVT) were more common in tamoxifen-containing strategies. In contrast, rates of osteoporosis, fracture and arthralgia were higher with OFS + AI compared to OFS + tamoxifen and tamoxifen. Rates of hot flushes and hypertension did not differ between the OFS regimens and were higher than tamoxifen monotherapy (Supplementary Table S3). Pooled discontinuation rates were 19.3% for tamoxifen, 18.3% for OFS + tamoxifen, and 23.7% for OFS + AI. Similar patterns were noted for the AI versus tamoxifen comparison in postmenopausal women, while AI for five years was associated with a lower risk of DVT and higher risk of osteoporosis than switch strategy (Supplementary Table S4). Pooled discontinuation rates were 13.2% for tamoxifen, 16.2% for switch strategy, and 11.2% for AI.

### Extended endocrine therapy following five years of tamoxifen

#### Trial-level network meta-analysis

**Fig. 4:**
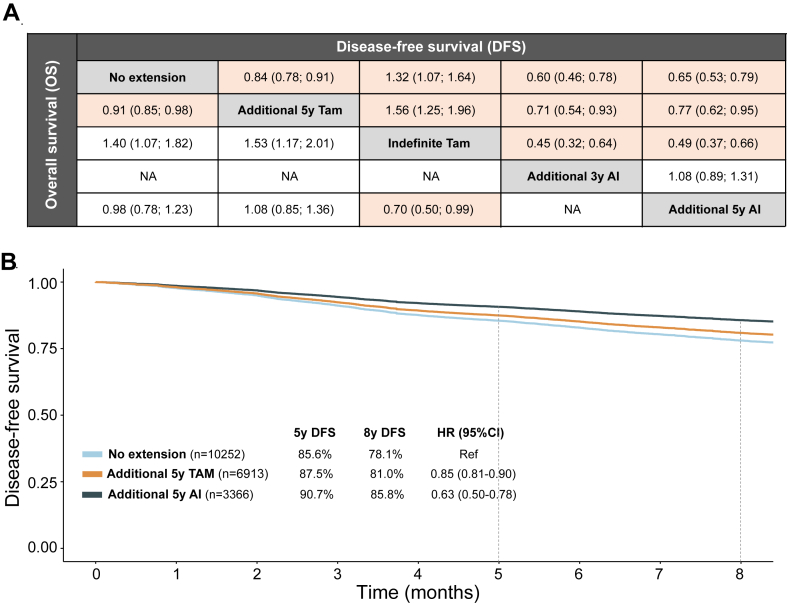
Comparison of treatment strategies following five years of tamoxifen. A) trial-level network meta-analysis. Disease-free and overall survival are represented by means of hazard ratios. The hazard ratios for comparisons are in the cell in common between the column and row treatment, a hazard ratio lower than 1 favor column-defining treatment. The statistically significant comparisons are represented in color. B) Kaplan–Meier curves for disease-free survival, generated with extracted individual patient data, comparing three treatment strategies of no extended therapy, additional five years of tamoxifen and additional five years of aromatase inhibitors. Abbreviations: DFS: disease-free survival; TAM: tamoxifen; AI: aromatase inhibitor; HR: hazard ratio; CI: confidence interval.

Fig. 2C presents the network graph of treatment strategies following five years of tamoxifen and Fig. 4A summarizes the results of the comparisons between five treatment strategies (n = 26,380): i) no further treatment, ii) additional five years of tamoxifen, iii) additional indefinite tamoxifen, iv) additional three years of AI, and v) additional five years of AI. Extended five years of tamoxifen and AI strategies were associated with improved DFS compared to tamoxifen for only five years. Importantly, extended AI for three years (HR = 0.71, 95% CI 0.54–0.93) and AI for five years (HR = 0.77, 95% CI 0.62–0.95) improved DFS compared with extended treatment with five years of tamoxifen. Finally, DFS did not differ in the comparison of extended AI for three and AI for five years (HR = 1.08, 95% CI 0.89–1.31). For the endpoint of OS, three additional years of AI could not be compared with other strategies due to paucity of data. The only strategy that was statistically significantly associated with improved OS over 5 years of tamoxifen was extended treatment with 5 more years of tamoxifen (HR = 0.91, 95% CI 0.85–0.98).

#### Extracted individual patient data meta-analysis

Three treatment strategies with available Kaplan–Meier curves in the respective publications were compared: i) no extended therapy, ii) additional five years of tamoxifen, and iii) additional five years of AI (Fig. 4B). Both extended therapies improved DFS compared to no extended therapy with ≥4% DFS benefit at 8-years. In the comparison between AI and tamoxifen extended strategies, the AI strategy was associated with numerically higher DFS rates at 5-years (90.7% vs 87.5%) and at 8-years (85.8% vs 81.0%; NNT 15 for disease relapse at 8 years).

#### Safety

Compared to no extended therapy, continuation with 5 years of AI was associated with increased risk of osteoporosis, fracture, arthralgia and hot flushes. Continuation with 5 years of tamoxifen was associated with an increased risk of DVT compared to no extended therapy (Supplementary Table S5).

### Extended endocrine therapy following five years of aromatase inhibitor or switch strategy

#### Trial-level network meta-analysis

We first compared any extended therapy with AI regardless of duration, following treatment for five years with AI or switch strategy (Supplementary Figure S5). Extended ET was associated with improved DFS in the entire population (HR = 0.81, 95% CI 0.73–0.90) and consistently across subgroups defined by nodal status, (neo)adjuvant chemotherapy and prior ET. Notably, only patients with PR positive breast cancer seemed to benefit from extended AI (HR = 0.69, 95% CI 0.55–0.87) but not those with PR negative disease (HR = 1.04, 95% CI 0.52–2.09). The association between extended ET and OS did not reach statistical significance (HR = 0.92, 95% CI 0.82–1.03), with wide CI in all subgroups and a clearer signal in patients previously exposed to both tamoxifen and AI (HR = 0.78, 95% CI 0.61–1.00).

**Fig. 5:**
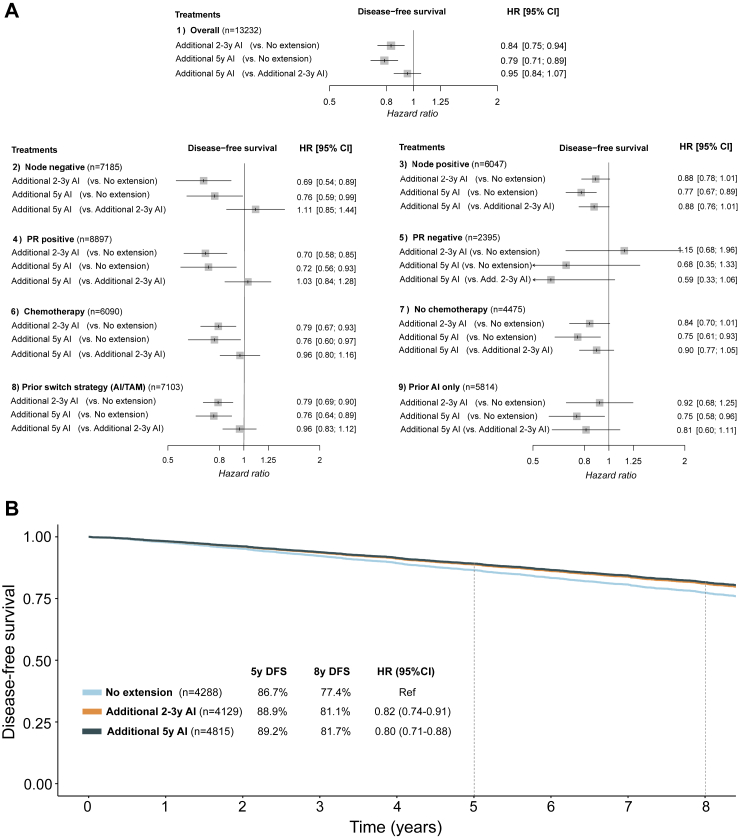
Comparison of treatment strategies following five years of aromatase inhibitor or switch strategy. A) trial-level network meta-analysis. Comparisons in terms of disease-free survival in entire population (A1) and according to nodal status (A2), progesterone receptor status (A3), receipt of (neo)adjuvant chemotherapy (A4), and type of prior endocrine treatment (A5). B) Kaplan–Meier curves for disease-free survival, generated with extracted individual patient data, comparing three treatment strategies of no extended therapy, additional two to three years and additional five years of aromatase inhibitors. Abbreviations: DFS: disease-free survival; TAM: tamoxifen; AI: aromatase inhibitor; HR: hazard ratio; CI: confidence interval; PR: progesterone receptor.

We then compared different treatment lengths of extended treatment with AI. Fig. 2D presents the network graph of treatment strategies following five years of AI or AI and tamoxifen and Fig. 5A summarizes the results of three treatment strategies (n = 13,232): i) no further treatment, ii) additional two to three years of AI, and iii) additional five years of AI. In the total population and across subgroups defined by nodal status and receipt of chemotherapy, both extended endocrine treatment strategies improved DFS compared to treatment discontinuation at five years. An additional five years of AI consistently improved DFS in all subgroups compared to no extended treatment, while compared to two to three years of AI, the point estimates for DFS were in favor of additional five years mostly in patients with prior AI only (HR = 0.81, 95% CI 0.60–1.11), PR-negative disease (HR = 0.59, 95% CI 0.33–1.06), and node positive disease (HR = 0.88, 95% CI 0.76–1.01), albeit with wide CI in all subgroups.

For the OS endpoint, only shorter duration of extension was associated with borderline improved outcomes compared to no extension (HR = 0.86, 95% CI 0.73–1.01), but not longer duration (HR = 0.98, 95% CI 0.83–1.15). This improvement was most clearly observed in patients with PR positive breast cancer and in those previously treated with switch strategy, although few events and wide CI were noted in these subgroup analyses (Supplementary Figure S6).

#### Extracted individual patient data meta-analysis

In the overall population, the corresponding 8-year DFS rates with no further treatment were 77.4%, with two to three years of AI 81.1% and with five years of AI 81.7% (NNT 24) according to reconstructed individual patient data. Both extended treatment strategies improved DFS compared with no extended treatment (two to three years, HR = 0.82, 95% CI 0.74–0.91 and five years, HR = 0.80, 95% CI 0.71–0.88) (Fig. 5B).

#### Safety

Compared to no extended therapy, continuation with AI for five years was associated with higher pooled rates of osteoporosis, fracture, arthralgia, and hot flushes. Shorter extended therapy was associated with higher risk of arthralgia and hot flushes compared to no extended therapy, while no statistically significant differences were observed between the two extended ET strategies (Supplementary Table S6). Pooled discontinuation rates were 23.2% for five additional years with AI, 29.6% for two to three additional years, and 17.6% for placebo.

### Addition of targeted agents

#### Trial-level network meta-analysis

Fig. 2E presents the network graph of the addition of targeted agents and Fig. 5A shows the results of the two strategies that were compared to ET alone (n = 20,789): i) addition of a cyclin dependent kinases 4/6 inhibitor (CDK4/6i) and ii) addition of a mammalian target of rapamycin inhibitor (mTORi). The former improved DFS in the entire population (HR = 0.79, 95% CI 0.67–0.94) and in all subgroups according to menopausal status, nodal status and timing of chemotherapy. In contrast, addition of everolimus was not associated with improved DFS, except for premenopausal patients (HR = 0.64, 95% CI 0.43–0.96) (Supplementary Figure S7A). Neither mTORi nor CDK4/6i were associated with improved OS over ET alone, although follow-up in these studies was short (HR = 1.00, 95% CI 0.73–1.37 and HR = 0.96, 95% CI 0.79–1.17, respectively; Supplementary Figure S7).

#### Extracted individual patient data meta-analysis

At four years, combining CDK4/6i with ET was associated with an improvement of 3.6% in the DFS rate compared to ET alone, 85.5% vs 81.9% respectively (HR = 0.78, 95% CI 0.72–0.85; Fig. 5B), resulting in a NNT of 30.

#### Safety

Addition of CDK4/6i was associated with increased rates of any grade 3 adverse event (74.8% vs 14.6%), grade 3 or worse neutropenia (43.9% vs 0.7%), diarrhea (37.4% vs 8%), nausea (24.4% vs 10.8%) compared to ET alone, but with lower reported rates of hot flushes (22.5% vs 28.3%) and arthralgia (34.2% vs 41.2%).

## Discussion

Current guidelines recommend ET for almost all patients with hormone receptor positive tumors.^61^, ^62^, ^63^ Beyond this broad recommendation specific guidance is limited, with some guidelines suggesting that treatment escalation for premenopausal women, or extended treatment with one of many studied regimens, should be considered.^62^ Large RCT and individual patient data meta-analyses provide guidance regarding the management of specific patient subgroups. However, these approaches are limited to previously conducted direct comparisons. By performing a network meta-analysis of 37 RCT that had enrolled more than 107,000 patients we provide additional evidence by reporting on indirect comparisons between different ET strategies not previously compared with each other, and by increasing sample size and thus precision of estimates of direct comparisons.

Firstly, we expanded on the results of the SOFT and TEXT trials,^49^^,^^50^ as with large statistical power we demonstrate the superiority of OFS + AI over tamoxifen monotherapy even for patients with lower risk of recurrence, such as those with node negative disease or those not treated with chemotherapy, which was not addressed by the EBCTCG meta-analysis of OFS + AI vs OFS + Tamoxifen.^3^ Moreover, while several RCT have shown that continuing treatment after five years of tamoxifen with either tamoxifen or AI improves outcomes, no direct comparison between these two strategies has been performed. Our findings suggest that switching to an AI may be the superior strategy for these patients. Lastly, our analysis on the optimal approach to patients following five years of AI or switch strategy revealed that patients with node-positive breast cancer may potentially benefit more from extending AI therapy for an additional five years. Conversely, for most patients considered for extended ET an additional two to three years of AI is sufficient. This conclusion diverges from two published RCT which could not identify any subgroup benefiting from more extended treatment.^34^^,^^37^

A secondary aim of this study was to quantify the absolute risk reduction associated with various ET strategies. Results of trial-level meta-analyses are commonly reported as relative risk reduction, which is less intuitive and more difficult for patients to understand and physicians to put into context for the individual patient, in relation to the expected absolute benefit and adverse events. In our study, the absolute benefit in terms of recurrence by evaluated strategies of ET escalation varied considerably, with an associated NNT ranging from 13 to 35 for unselected patients, depending on the strategy (Supplementary Table S7). While these findings indicate some overtreatment and patient exposure to unnecessary side effects, the overall risk—benefit ratio of selected strategies is favorable for patients at higher risk of recurrence considering the low cost of ET, very low risk of fatal adverse events, long-term clinical experience with the agents, their widespread availability and established evidence-based strategies to mitigate side effects and improve tolerability.^64^ However, adverse events are commonly underreported in RCT,^65^ which also affects the results of our pooled safety analysis and leads to potential underestimation of the associated risks with ET. This observation, in addition to the considerable discontinuation rates of ET owing to toxicity and worsening of quality of life,^66^^,^^67^ are crucial factors that need to be considered in parallel with the expected absolute risk reduction, which for patients at low risk for recurrence may not be sufficient to motivate escalated ET.

Strengths of our study include its broad scope that encompasses all clinical scenarios and treatment strategies previously reported in RCT, the large sample size which allowed for more precise subgroup analyses, and the long follow-up period since most studies, except for those reporting on targeted agents, have reported end-of-study results. However, our study has limitations, including the heterogeneity of the studied regimens, the lack of original individual patient data which did not allow analysis of more complex subgroups with different combinations of prognostic factors and the intrinsic limitations of the available evidence. For example, most RCT have enrolled postmenopausal patients and guidance on extended ET for premenopausal ones following five years of OFS + AI is extrapolated from such studies. Since no trials have evaluated OFS beyond five years, the management of patients remaining premenopausal following the first five years should be individualized. In addition, gene expression profiling has emerged as a tool that predicts benefit from extended ET. However, not all “prospective-retrospective” analyses have shown a significant interaction between risk classification and treatment benefit,^68^^,^^69^ there is no confirmatory prospective RCT and these tools are not available in all regions and practices, observations that underscore the clinical utility of our findings. Furthermore, heterogeneity between different trials in the precise endpoint definitions, methods of assessment of ER and PR expression, and reporting of adverse events could be a confounding factor to our results. A particular mention should be made about the cut-off for ER positivity, thus the definition of the candidate population for ET. While molecular,^70^ immunologic,^71^ and clinical data^72^^,^^73^ indicate that “ER-low” may represent ER-negative disease, a recent retrospective study demonstrated benefit from ET in this population, highlighting the need for a randomized trial specific for this population, assessed with contemporary methods for ER expression and otherwise treated with contemporary local and systemic therapy. Finally, there is uncertainty concerning the benefit from CDK4/6i, as there might be differences in efficacy among the three available agents although no direct comparisons have been made, while follow-up is still short, with no observed OS benefit.

In conclusion, this network meta-analysis summarizes the available evidence on adjuvant endocrine therapy for non-metastatic breast cancer. By extrapolating from the tamoxifen for five years versus no endocrine treatment EBCTCG overview,^1^ an optimal treatment strategy reduces the risk of recurrence by two thirds. The proportional risk reduction should however be considered in relation to the risk for adverse events and the expected absolute benefit from escalated ET strategies, since statistical and clinical significance are not synonymous, and some patients may derive minimal absolute benefit from ET. Results from individual patient data meta-analyses will shed further light on the management of specific patient subgroups.

## Contributors

Concept and design: Papakonstantinou, Villacampa, Matikas; Literature search: Papakonstantinou, Matikas; Data extraction: Papakonstantinou, Matikas; Statistical analysis: Villacampa, Navarro; Interpretation of data: all authors; Administrative, technical, or material support: Pascual, Matikas; Writing of initial draft: Papakonstantinou, Villacampa, Matikas; Critical revisions and approval of final draft: all authors. All authors read and approved the final version of the manuscript. Papakonstantinou, Villacampa and Matikas had full access to the underlying data.

## Data sharing statement

Published data from clinical trials were used in the analyses which will be made available upon a written request to the corresponding author.

## Declaration of interests

Guillermo Villacampa: speaker's fee from Pfizer, MSD, GSK and Pierre Fabrer; advisory role with AstraZeneca; consultant fees from Reveal Genomics. Mafalda Oliveira: consulting fees from Roche, Seagen, GlaxoSmithKline, Gilead, Puma Biotechnology, AstraZeneca, iTeos Therapeutics, Pierre Fabre, and MSD; research funding from AstraZeneca, Genentech, Roche, Novartis, Immunomedics, Seagen, GlaxoSmithKline, Boehringer Ingelheim, Puma Biotechnology, and Zenith Epigenetics; honoraria from Roche, Seagen, Novartis, AstraZeneca, and Eisai; travel grants from Roche, Pierre Fabre, Novartis, and Eisai; Antonis Valachis: unrestricted research funding paid to institution by Roche and MSD. Tomas Pascual: speaker's fee from Pfizer, AstraZeneca, Novartis, Veracyte and Argenetics; advisory role with Novartis; Alexios Matikas: speaker/consultancy (no personal fees) to Veracyte, Roche, Seagen; research funding paid to institution by MSD, AstraZeneca, Novartis, Veracyte. Andri Papakonstantinou and Victor Navarro have no conflicts of interest to declare.
