# Supplementary material for: Adjuvant endocrine treatment strategies for non-metastatic breast cancer: a network meta-analysis
Source: eClinicalMedicine. 2025 Feb 17;81:103116. doi: 10.1016/j.eclinm.2025.103116 (PMC11875833; doi:10.1016/j.eclinm.2025.103116)

**Data Supplement to**

**Endocrine treatment strategies for non-metastatic breast cancer: a network meta-analysis**

**Table of Contents**

**Supplementary Search Strategies**………………………………………………………………………………**2**

**Supplementary Tables**……………………………………………………………………………………………….

**Table S1.** Definition of primary endpoint of included trials**15**

**Table S2.** Randomized trials that were excluded from the meta-analysis **19**

**Table S3.** Premenopausal patients, endocrine treatment during first five years: safety**20**

**Table S4.** Postmenopausal patients, endocrine treatment during first five years: safety **21**

**Table S5.** Extended therapy following tamoxifen for five years: safety **22**

**Table S6.** Extended therapy following tamoxifen or AI for five years: safety **23**

**Table S7.** Numbers needed to treat to avoid one relapse**24**

**Supplementary Figures**…………………………………………………………………………………………….

**Figure S1.** Geographic distribution of included randomized trials**25**

**Figure S2.** Risk of bias**26**

**Figure S3.** Consistency of the network estimates**27**

**Figure S4.** OS analysis of endocrine treatment during the first five years**28**

**Figure S5.** DFS and OS analysis of any extended therapy after AI or switch strategy**29**

**Figure S6.** OS analysis of extended therapy following AI or switch strategy**30**

**Figure S7.** DFS analysis of the addition of molecularly targeted agents**31**

**Figure S8.** OS analysis of the addition of molecularly targeted agents**32**

Documentation of search strategies

University Library search consultation group

Date: Original Search 19 September 2023, latest update 26 August 2024

Topic/research question: Adjuvant endocrine therapy for breast cancer

Name of researcher(s): Alexios Matikas

Librarian(s): Emma-Lotta Säätelä & Sabina Gillsund

Databases:

1. Medline (Ovid)
2. Embase (embase.com)
3. Cochrane Library (Wiley)
4. Web of Science Core Collection (Clarivate)

Total number of hits:

- Before deduplication: 29,955
- After deduplication: 17,673

Reports assessed for eligibility

(n = 1)

**Identification of studies via databases and registers**

**Identification of studies via other methods**

Records removed *before screening*:

Duplicate records removed (n =12 282)

Records marked as ineligible by automation tools (n = )

Records removed for other reasons (n = )

Records identified from*:

Databases (n=29 955

- Medline n =8 162
- Embase=7 858
- Cochrane=7 446
- WoS=6 489)

Registers (n = )

Records screened

(n = 17 673)

Records excluded**

(n =17 435 )

Reports sought for retrieval

(n = 1)

Reports sought for retrieval

(n = 238)

Reports not retrieved

(n = )

**Screening**

Reports excluded:

Reason 1 (n = )

Reason 2 (n = )

Reason 3 (n = )

etc.

Reports assessed for eligibility

(n = 78)

Reports excluded:

Not fulfilling eligibility criteria (n =6)

Multiple publications from same study (n=36)

Records identified from:

Websites (n = 1)

Organisations (n = )

Citation searching (n = )

etc.

**Identification**

Reports not retrieved

(n = )

**Included**

Studies included in review

(n = 37)

Reports of included studies

(n = )

*From:*  Page MJ, McKenzie JE, Bossuyt PM, Boutron I, Hoffmann TC, Mulrow CD, et al. The PRISMA 2020 statement: an updated guideline for reporting systematic reviews. BMJ 2021;372:n71. doi: 10.1136/bmj.n71. For more information, visit: <http://www.prisma-statement.org/>

1. Medline

| Interface: Ovid MEDLINE(R) ALL  Date of Search: August 26, 2024  Number of hits: 8,162  Comment: In Ovid, two or more words are automatically searched as phrases; i.e. no quotation marks are needed | Field labels   - exp/ = exploded MeSH term - / = non exploded MeSH term - .ti,ab,kf. = title, abstract and author keywords - adjx = within x words, regardless of order - * = truncation of word for alternate endings |
| --- | --- |
| Database(s): **Ovid MEDLINE(R) ALL**1946 to August 23, 2024 Search Strategy:   \| **#** \| **Searches** \| **Results** \| \| --- \| --- \| --- \| \| 1 \| exp Breast neoplasms/ \| 357 491 \| \| 2 \| ((breast or mammary) adj3 (adenocarcinoma* or cancer* or carcinoma* or malign* or neoplasm* or sarcoma* or tumour* or tumor*)).ti,ab,kf. \| 450 370 \| \| 3 \| 1 or 2 \| 514 992 \| \| 4 \| Antineoplastic Agents, Hormonal/ \| 17 836 \| \| 5 \| Aromatase inhibitors/ \| 7 070 \| \| 6 \| Selective Estrogen Receptor Modulators/ \| 4 335 \| \| 7 \| Triazoles/ \| 27 131 \| \| 8 \| Anastrozole/ \| 1 575 \| \| 9 \| Letrozole/ \| 2 703 \| \| 10 \| Tamoxifen/ \| 20 688 \| \| 11 \| Nitriles/ \| 30 258 \| \| 12 \| exp Gonadotropin-Releasing Hormone/ \| 34 989 \| \| 13 \| (endocrin* adj3 (therap* or treatment*)).ti,ab,kf. \| 15 946 \| \| 14 \| (hormon* adj3 (antineoplas* or anti-neoplas*)).ti,ab,kf. \| 72 \| \| 15 \| aromatase inhibitor*.ti,ab,kf. \| 9 095 \| \| 16 \| (selective estrogen receptor modulator? or SERM?).ti,ab,kf. \| 4 476 \| \| 17 \| triazole*.ti,ab,kf. \| 16 590 \| \| 18 \| (anastr#zole or arimidex or ICI D1033 or ZD-1033 or ZD1033 or femathina or ici d1033 or icid1033 or mpi 674 or mpi 676 or mpi674 or mpi676 or trozolet or zd 1033 or zd1033).ti,ab,kf. \| 2 295 \| \| 19 \| (CGS20267 or CGS 20267 or femar? or loxifan or letrozol*).ti,ab,kf. \| 4 224 \| \| 20 \| (aromasi* or ex#mestane or fce24304 or nakides or nikidess or pnu 155971 or pnu155971 or fce 24304).ti,ab,kf. \| 1 583 \| \| 21 \| (ebefen or ici46474 or ici47699 or ici-46474 or ici-47699 or kessar or nolvadex or novaldex or nsc 180973 or pt101 or pt 101 or soltamox or tamoplac or tamoxasta or tamoxifen? or tomaxithen or zitazonium).ti,ab,kf. \| 26 362 \| \| 22 \| nitrile?.ti,ab,kf. \| 8 890 \| \| 23 \| (ovar* adj4 suppress*).ti,ab,kf. \| 3 789 \| \| 24 \| (cystorelin or dirigestran or factrel or fsh releasing hormone or gn-rh or gnrh or gonadoliberin or gonadorelin or gonadotropin releasing hormone or kryptocur or lfrh or lh-releasing hormone or lh-rh or lhfsh releasing hormone or lhfshrh or lhrh or luliberin or luteinizing hormone-releasing).ti,ab,kf. \| 42 394 \| \| 25 \| (goserelin or ici-118630 or ici118630 or zoladex).ti,ab,kf. \| 1 354 \| \| 26 \| (ay25650 or ay-25650 or cl118532 or cl-118532 or d-trp-6 or debio 8200 or debio 8206 or debio8200 or debio8206 or detryptorelin or diphereline or fertipeptil or gonapeptyl or "isr 048" or isr 48 or isr048 or isr48 or decapeptyl or "ly 01007" or ly01007 or microrelin or moapar or ovugel or pamorelin or salvacyl or spherotide or triptodur or triptofem or tr#ptorelin? or trelstar or wy42462 or wy-42462).ti,ab,kf. \| 1 273 \| \| 27 \| or/4-26 \| 180 972 \| \| 28 \| 3 and 27 \| 34 925 \| \| 29 \| randomized controlled trial.pt. \| 619 647 \| \| 30 \| controlled clinical trial.pt. \| 95 592 \| \| 31 \| randomi#ed.ti,ab. \| 851 589 \| \| 32 \| placebo.ti,ab. \| 257 895 \| \| 33 \| randomly.ti,ab. \| 441 425 \| \| 34 \| trial.ti,ab. \| 823 123 \| \| 35 \| groups.ti,ab. \| 2 752 873 \| \| 36 \| or/29-35 \| 4 012 460 \| \| 37 \| (animals not humans).sh. \| 5 217 352 \| \| 38 \| 36 not 37 \| 3 485 634 \| \| 39 \| 28 and 38 \| 8 267 \| \| 40 \| limit 39 to (editorial or letter or preprint) \| 105 \| \| **41** \| **39 not 40** \| **8 162** \| | |

Embase

| Interface: embase.com  Date of Search: August 26, 2024  Number of hits: 7,858  Comment: Emtree is the controlled vocabulary in Embase | Field labels   - /exp = exploded Emtree term - /de = non exploded Emtree term - /mj = major Emtree term - ti,ab,kw = title, abstract and author keywords - NEAR/x = within x words, regardless of order - * = truncation of word for alternate endings |
| --- | --- |
| \| **#** \| **Searches** \| **Results** \| \| --- \| --- \| --- \| \| #01 \| 'breast cancer'/exp \| 619 105 \| \| #02 \| ((breast OR mammary) NEAR/2 (adenocarcinoma* OR cancer* OR carcinoma* OR malign* OR neoplasm* OR sarcoma* OR tumour* OR tumor*)):ti,ab,kw \| 614 470 \| \| #03 \| #1 OR #2 \| 760 880 \| \| #04 \| 'cancer hormone therapy'/exp/mj \| 5 809 \| \| #05 \| 'aromatase inhibitor'/exp/mj \| 12 881 \| \| #06 \| 'selective estrogen receptor modulator'/mj \| 2 110 \| \| #07 \| 'triazole derivative'/mj \| 5 561 \| \| #08 \| 'tamoxifen'/mj \| 19 593 \| \| #09 \| 'nitrile'/mj \| 6 687 \| \| #10 \| 'gonadorelin derivative'/mj OR 'gonadorelin agonist'/exp/mj OR 'goserelin'/exp/mj OR 'triptorelin'/exp/mj \| 14 111 \| \| #11 \| (endocrin* NEAR/2 (therap* OR treatment*)):ti,ab,kw \| 25 634 \| \| #12 \| (hormon* NEAR/2 (antineoplas* OR 'anti neoplas*')):ti,ab,kw \| 109 \| \| #13 \| 'aromatase inhibitor*':ti,ab,kw \| 15 468 \| \| #14 \| 'selective estrogen receptor modulator$':ti,ab,kw OR serm$:ti,ab,kw \| 6 803 \| \| #15 \| triazole*:ti,ab,kw \| 20 293 \| \| #16 \| anastr?zole:ti,ab,kw OR arimidex:ti,ab,kw OR femathina:ti,ab,kw OR 'ici d1033':ti,ab,kw OR icid1033:ti,ab,kw OR 'mpi 674':ti,ab,kw OR 'mpi 676':ti,ab,kw OR mpi674:ti,ab,kw OR mpi676:ti,ab,kw OR trozolet:ti,ab,kw OR 'zd 1033':ti,ab,kw OR zd1033:ti,ab,kw \| 4 229 \| \| #17 \| cgs20267:ti,ab,kw OR 'cgs 20267':ti,ab,kw OR femar$:ti,ab,kw OR loxifan:ti,ab,kw OR letrozol*:ti,ab,kw \| 8 180 \| \| #18 \| aromasi*:ti,ab,kw OR ex?mestane:ti,ab,kw OR fce24304:ti,ab,kw OR nakides:ti,ab,kw OR nikidess:ti,ab,kw OR 'pnu 155971':ti,ab,kw OR pnu155971:ti,ab,kw OR 'fce 24304':ti,ab,kw \| 3 093 \| \| #19 \| ebefen:ti,ab,kw OR ici46474:ti,ab,kw OR ici47699:ti,ab,kw OR 'ici 46474':ti,ab,kw OR 'ici 47699':ti,ab,kw OR kessar:ti,ab,kw OR nolvadex:ti,ab,kw OR novaldex:ti,ab,kw OR 'nsc 180973':ti,ab,kw OR pt101:ti,ab,kw OR 'pt 101':ti,ab,kw OR soltamox:ti,ab,kw OR tamoplac:ti,ab,kw OR tamoxasta:ti,ab,kw OR tamoxifen$:ti,ab,kw OR tomaxithen:ti,ab,kw OR zitazonium:ti,ab,kw \| 40 849 \| \| #20 \| nitrile$:ti,ab,kw \| 11 225 \| \| #21 \| (ovar* NEAR/3 suppress*):ti,ab,kw \| 4 172 \| \| #22 \| cystorelin:ti,ab,kw OR dirigestran:ti,ab,kw OR factrel:ti,ab,kw OR 'fsh releasing hormone':ti,ab,kw OR 'gn rh':ti,ab,kw OR gnrh:ti,ab,kw OR gonadoliberin:ti,ab,kw OR gonadorelin:ti,ab,kw OR 'gonadotropin releasing hormone':ti,ab,kw OR kryptocur:ti,ab,kw OR lfrh:ti,ab,kw OR 'lh-releasing hormone':ti,ab,kw OR 'lh rh':ti,ab,kw OR 'lhfsh releasing hormone':ti,ab,kw OR lhfshrh:ti,ab,kw OR lhrh:ti,ab,kw OR luliberin:ti,ab,kw OR 'luteinizing hormone-releasing':ti,ab,kw \| 56 298 \| \| #23 \| goserelin:ti,ab,kw OR 'ici 118630':ti,ab,kw OR ici118630:ti,ab,kw OR zoladex:ti,ab,kw \| 2 144 \| \| #24 \| ay25650:ti,ab,kw OR 'ay 25650':ti,ab,kw OR cl118532:ti,ab,kw OR 'cl 118532':ti,ab,kw OR 'd trp 6':ti,ab,kw OR 'debio 8200':ti,ab,kw OR 'debio 8206':ti,ab,kw OR debio8200:ti,ab,kw OR debio8206:ti,ab,kw OR detryptorelin:ti,ab,kw OR diphereline:ti,ab,kw OR fertipeptil:ti,ab,kw OR gonapeptyl:ti,ab,kw OR 'isr 048':ti,ab,kw OR 'isr 48':ti,ab,kw OR isr048:ti,ab,kw OR isr48:ti,ab,kw OR decapeptyl:ti,ab,kw OR 'ly 01007':ti,ab,kw OR ly01007:ti,ab,kw OR microrelin:ti,ab,kw OR moapar:ti,ab,kw OR ovugel:ti,ab,kw OR pamorelin:ti,ab,kw OR salvacyl:ti,ab,kw OR spherotide:ti,ab,kw OR triptodur:ti,ab,kw OR triptofem:ti,ab,kw OR tr?ptorelin$:ti,ab,kw OR trelstar:ti,ab,kw OR wy42462:ti,ab,kw OR 'wy 42462':ti,ab,kw \| 2 402 \| \| #25 \| #4 OR #5 OR #6 OR #7 OR #8 OR #9 OR #10 OR #11 OR #12 OR #13 OR #14 OR #15 OR #16 OR #17 OR #18 OR #19 OR #20 OR #21 OR #22 OR #23 OR #24 \| 184 372 \| \| #26 \| #3 AND #25 \| 53 635 \| \| #27 \| ('randomized controlled trial'/de OR 'controlled clinical trial'/de OR random*:ti,ab,tt OR 'randomization'/de OR 'intermethod comparison'/de OR placebo:ti,ab,tt OR compare:ti,tt OR compared:ti,tt OR comparison:ti,tt OR ((evaluated:ab OR evaluate:ab OR evaluating:ab OR assessed:ab OR assess:ab) AND (compare:ab OR compared:ab OR comparing:ab OR comparison:ab)) OR ((open NEXT/1 label):ti,ab,tt) OR (((double OR single OR doubly OR singly) NEXT/1 (blind OR blinded OR blindly)):ti,ab,tt) OR 'double blind procedure'/de OR ((parallel NEXT/1 group*):ti,ab,tt) OR crossover:ti,ab,tt OR 'cross over':ti,ab,tt OR (((assign* OR match OR matched OR allocation) NEAR/6 (alternate OR group OR groups OR intervention OR interventions OR patient OR patients OR subject OR subjects OR participant OR participants)):ti,ab,tt) OR assigned:ti,ab,tt OR allocated:ti,ab,tt OR ((controlled NEAR/8 (study OR design OR trial)):ti,ab,tt) OR volunteer:ti,ab,tt OR volunteers:ti,ab,tt OR 'human experiment'/de OR trial:ti,tt) NOT (((random* NEXT/1 sampl* NEAR/8 ('cross section*' OR questionnaire* OR survey OR surveys OR database OR databases)):ti,ab,tt) NOT ('comparative study'/de OR 'controlled study'/de OR 'randomised controlled':ti,ab,tt OR 'randomized controlled':ti,ab,tt OR 'randomly assigned':ti,ab,tt) OR ('cross‐sectional study' NOT ('randomized controlled trial'/de OR 'controlled clinical study'/de OR 'controlled study'/de OR 'randomised controlled':ti,ab,tt OR 'randomized controlled':ti,ab,tt OR 'control group':ti,ab,tt OR 'control groups':ti,ab,tt)) OR ('case control*':ti,ab,tt AND random*:ti,ab,tt NOT ('randomised controlled':ti,ab,tt OR 'randomized controlled':ti,ab,tt)) OR ('systematic review':ti,tt NOT (trial:ti,tt OR study:ti,tt)) OR (nonrandom*:ti,ab,tt NOT random*:ti,ab,tt) OR 'random field*':ti,ab,tt OR (('random cluster' NEAR/4 sampl*):ti,ab,tt) OR (review:ab AND review:it NOT trial:ti,tt) OR ('we searched':ab AND (review:ti,tt OR review:it)) OR 'update review':ab OR ((databases NEAR/5 searched):ab) OR ((rat:ti,tt OR rats:ti,tt OR mouse:ti,tt OR mice:ti,tt OR swine:ti,tt OR porcine:ti,tt OR murine:ti,tt OR sheep:ti,tt OR lambs:ti,tt OR pigs:ti,tt OR piglets:ti,tt OR rabbit:ti,tt OR rabbits:ti,tt OR cat:ti,tt OR cats:ti,tt OR dog:ti,tt OR dogs:ti,tt OR cattle:ti,tt OR bovine:ti,tt OR monkey:ti,tt OR monkeys:ti,tt OR trout:ti,tt OR marmoset*:ti,tt) AND 'animal experiment'/de) OR ('animal experiment'/de NOT ('human experiment'/de OR 'human'/de))) \| 5 930 599 \| \| #28 \| #26 AND #27 \| 14 581 \| \| #29 \| #26 AND #27 AND ([conference abstract]/lim OR [conference review]/lim OR [editorial]/lim OR [letter]/lim OR [note]/lim OR [preprint]/lim) \| 6 728 \| \| **#30** \| **#28 NOT #29** \| **7 858** \| | |

3. Cochrane Library

| Interface: Wiley  Date of Search: August 26, 2024  Number of hits: 7,446 | Field labels   - ti,ab,kw = title, abstract and author keywords - NEAR/x = within x words, regardless of order - * = truncation of word for alternate endings |
| --- | --- |
| \| **#** \| **Searches** \| **Results** \| \| --- \| --- \| --- \| \| #1 \| [mh "Breast neoplasms"] \| 20 356 \| \| #2 \| ((breast:ti,ab,kw OR mammary:ti,ab,kw) NEAR/3 (adenocarcinoma*:ti,ab,kw OR cancer*:ti,ab,kw OR carcinoma*:ti,ab,kw OR malign*:ti,ab,kw OR neoplasm*:ti,ab,kw OR sarcoma*:ti,ab,kw OR tumour*:ti,ab,kw OR tumor*:ti,ab,kw)) \| 47 859 \| \| #3 \| #1 OR #2 \| 47 859 \| \| #4 \| [mh ^"Antineoplastic Agents, Hormonal"] \| 2 126 \| \| #5 \| [mh ^"Aromatase inhibitors"] \| 949 \| \| #6 \| [mh ^"Selective Estrogen Receptor Modulators"] \| 567 \| \| #7 \| [mh ^Triazoles] \| 2 039 \| \| #8 \| [mh ^Anastrozole] \| 566 \| \| #9 \| [mh ^Letrozole] \| 976 \| \| #10 \| [mh ^Tamoxifen] \| 2 373 \| \| #11 \| [mh ^Nitriles] \| 1 977 \| \| #12 \| [mh "Gonadotropin-Releasing Hormone"] \| 3 334 \| \| #13 \| (endocrin*:ti,ab,kw NEAR/3 (therap*:ti,ab,kw OR treatment*:ti,ab,kw)) \| 4 842 \| \| #14 \| (hormon*:ti,ab,kw NEAR/3 (antineoplas*:ti,ab,kw OR ("anti" NEXT neoplas*):ti,ab,kw)) \| 2 163 \| \| #15 \| ("aromatase" NEXT inhibitor*):ti,ab,kw \| 2 808 \| \| #16 \| (("selective estrogen receptor" NEXT modulator?):ti,ab,kw OR SERM?:ti,ab,kw) \| 334 \| \| #17 \| triazole*:ti,ab,kw \| 2 165 \| \| #18 \| (anastr?zole:ti,ab,kw OR arimidex:ti,ab,kw OR "ICI D1033":ti,ab,kw OR ZD-1033:ti,ab,kw OR ZD1033:ti,ab,kw OR femathina:ti,ab,kw OR "ici d1033":ti,ab,kw OR icid1033:ti,ab,kw OR "mpi 674":ti,ab,kw OR "mpi 676":ti,ab,kw OR mpi674:ti,ab,kw OR mpi676:ti,ab,kw OR trozolet:ti,ab,kw OR "zd 1033":ti,ab,kw OR zd1033:ti,ab,kw) \| 1 497 \| \| #19 \| (CGS20267:ti,ab,kw OR "CGS 20267":ti,ab,kw OR femar?:ti,ab,kw OR loxifan:ti,ab,kw OR letrozol*:ti,ab,kw) \| 2 767 \| \| #20 \| (aromasi*:ti,ab,kw OR ex?mestane:ti,ab,kw OR fce24304:ti,ab,kw OR nakides:ti,ab,kw OR nikidess:ti,ab,kw OR "pnu 155971":ti,ab,kw OR pnu155971:ti,ab,kw OR "fce 24304":ti,ab,kw) \| 1 055 \| \| #21 \| (ebefen:ti,ab,kw OR ici46474:ti,ab,kw OR ici47699:ti,ab,kw OR ici-46474:ti,ab,kw OR ici-47699:ti,ab,kw OR kessar:ti,ab,kw OR nolvadex:ti,ab,kw OR novaldex:ti,ab,kw OR "nsc 180973":ti,ab,kw OR pt101:ti,ab,kw OR "pt 101":ti,ab,kw OR soltamox:ti,ab,kw OR tamoplac:ti,ab,kw OR tamoxasta:ti,ab,kw OR tamoxifen?:ti,ab,kw OR tomaxithen:ti,ab,kw OR zitazonium:ti,ab,kw) \| 5 499 \| \| #22 \| nitrile?:ti,ab,kw \| 2 008 \| \| #23 \| (ovar*:ti,ab,kw NEAR/4 suppress*:ti,ab,kw) \| 625 \| \| #24 \| (cystorelin:ti,ab,kw OR dirigestran:ti,ab,kw OR factrel:ti,ab,kw OR "fsh releasing hormone":ti,ab,kw OR gn-rh:ti,ab,kw OR gnrh:ti,ab,kw OR gonadoliberin:ti,ab,kw OR gonadorelin:ti,ab,kw OR "gonadotropin releasing hormone":ti,ab,kw OR kryptocur:ti,ab,kw OR lfrh:ti,ab,kw OR "lh-releasing hormone":ti,ab,kw OR lh-rh:ti,ab,kw OR "lhfsh releasing hormone":ti,ab,kw OR lhfshrh:ti,ab,kw OR lhrh:ti,ab,kw OR luliberin:ti,ab,kw OR "luteinizing hormone-releasing":ti,ab,kw) \| 6 754 \| \| #25 \| (goserelin:ti,ab,kw OR ici-118630:ti,ab,kw OR ici118630:ti,ab,kw OR zoladex:ti,ab,kw) \| 1 283 \| \| #26 \| (ay25650:ti,ab,kw OR ay-25650:ti,ab,kw OR cl118532:ti,ab,kw OR cl-118532:ti,ab,kw OR d-trp-6:ti,ab,kw OR "debio 8200":ti,ab,kw OR "debio 8206":ti,ab,kw OR debio8200:ti,ab,kw OR debio8206:ti,ab,kw OR detryptorelin:ti,ab,kw OR diphereline:ti,ab,kw OR fertipeptil:ti,ab,kw OR gonapeptyl:ti,ab,kw OR "isr 048":ti,ab,kw OR "isr 48":ti,ab,kw OR isr048:ti,ab,kw OR isr48:ti,ab,kw OR decapeptyl:ti,ab,kw OR "ly 01007":ti,ab,kw OR ly01007:ti,ab,kw OR microrelin:ti,ab,kw OR moapar:ti,ab,kw OR ovugel:ti,ab,kw OR pamorelin:ti,ab,kw OR salvacyl:ti,ab,kw OR spherotide:ti,ab,kw OR triptodur:ti,ab,kw OR triptofem:ti,ab,kw OR tr?ptorelin?:ti,ab,kw OR trelstar:ti,ab,kw OR wy42462:ti,ab,kw OR wy-42462:ti,ab,kw) \| 305 \| \| #27 \| #4 OR #5 OR #6 OR #7 OR #8 OR #9 OR #10 OR #11 OR #12 OR #13 OR #14 OR #15 OR #16 OR #17 OR #18 OR #19 OR #20 OR #21 OR #22 OR #23 OR #24 OR #25 OR #26 \| 23 187 \| \| #28 \| #3 AND #27 \| 9 195 \| \| #29 \| #28 NOT (clinicaltrials or trialsearch):so \| 7 487 \| \| **#30** \| **#29 AND Filter: Trials** \| **7 446** \| | |

4. Web of Science Core Collection

| Interface: Clarivate Analytics  Editions = A&HCI , ESCI , SCI-EXPANDED , SSCI  Date of Search: August 26, 2024  Number of hits: 6,489 | Field labels   - TS/Topic = title, abstract, author keywords and Keywords Plus - NEAR/x = within x words, regardless of order - * = truncation of word for alternate endings   Note: the *Exact search*-function was used for all the searches |
| --- | --- |
| \| **#** \| **Searches** \| **Results** \| \| --- \| --- \| --- \| \| 1 \| TS=((breast OR mammary ) NEAR/2 (adenocarcinoma* OR cancer* OR carcinoma* OR malign* OR neoplasm* OR sarcoma* OR tumour* OR tumor* )) \| 707 052 \| \| 2 \| TS=(endocrin* NEAR/2 (therap* OR treatment* )) \| 19 432 \| \| 3 \| TS=(hormon* NEAR/2 (antineoplas* OR anti-neoplas* )) \| 93 \| \| 4 \| TS="aromatase inhibitor*" \| 12 612 \| \| 5 \| TS=("selective estrogen receptor modulator$" OR SERM$ ) \| 5 185 \| \| 6 \| TS=triazole* \| 41 588 \| \| 7 \| TS=(anastr?zole OR arimidex OR "ICI D1033" OR ZD-1033 OR ZD1033 OR femathina OR "ici d1033" OR icid1033 OR "mpi 674" OR "mpi 676" OR mpi674 OR mpi676 OR trozolet OR "zd 1033" OR zd1033 ) \| 4 239 \| \| 8 \| TS=(CGS20267 OR "CGS 20267" OR femar$ OR loxifan OR letrozol* ) \| 7 231 \| \| 9 \| TS=(aromasi* OR ex?mestane OR fce24304 OR nakides OR nikidess OR "pnu 155971" OR pnu155971 OR "fce 24304" ) \| 2 615 \| \| 10 \| TS=(ebefen OR ici46474 OR ici47699 OR ici-46474 OR ici-47699 OR kessar OR nolvadex OR novaldex OR "nsc 180973" OR pt101 OR "pt 101" OR soltamox OR tamoplac OR tamoxasta OR tamoxifen$ OR tomaxithen OR zitazonium ) \| 39 355 \| \| 11 \| TS=nitrile$ \| 36 697 \| \| 12 \| TS=(ovar* NEAR/3 suppress* ) \| 4 104 \| \| 13 \| TS=(cystorelin OR dirigestran OR factrel OR "fsh releasing hormone" OR gn-rh OR gnrh OR gonadoliberin OR gonadorelin OR "gonadotropin releasing hormone" OR kryptocur OR lfrh OR "lh-releasing hormone" OR lh-rh OR "lhfsh releasing hormone" OR lhfshrh OR lhrh OR luliberin OR "luteinizing hormone-releasing" ) \| 52 959 \| \| 14 \| TS=(goserelin OR ici-118630 OR ici118630 OR zoladex ) \| 1 880 \| \| 15 \| TS=(ay25650 OR ay-25650 OR cl118532 OR cl-118532 OR d-trp-6 OR "debio 8200" OR "debio 8206" OR debio8200 OR debio8206 OR detryptorelin OR diphereline OR fertipeptil OR gonapeptyl OR "isr 048" OR "isr 48" OR isr048 OR isr48 OR decapeptyl OR "ly 01007" OR ly01007 OR microrelin OR moapar OR ovugel OR pamorelin OR salvacyl OR spherotide OR triptodur OR triptofem OR tr?ptorelin$ OR trelstar OR wy42462 OR wy-42462 ) \| 1 517 \| \| 16 \| #15 OR #14 OR #13 OR #12 OR #11 OR #10 OR #9 OR #8 OR #7 OR #6 OR #5 OR #4 OR #3 OR #2 \| 200 109 \| \| 17 \| #16 AND #1 \| 44 301 \| \| 18 \| TS=(randomised OR randomized OR randomisation OR randomization OR placebo* OR (random* AND (allocat* OR assign*) ) OR (blind* AND (single OR double OR treble OR triple) )) NOT TS=(animal or animals or pisces or fish or fishes or catfish or catfishes or sheatfish or silurus or arius or heteropneustes or clarias or gariepinus or fathead minnow or fathead minnows or pimephales or promelas or cichlidae or trout or trouts or char or chars or salvelinus or salmo or oncorhynchus or guppy or guppies or millionfish or poecilia or goldfish or goldfishes or carassius or auratus or mullet or mullets or mugil or curema or shark or sharks or cod or cods or gadus or morhua or carp or carps or cyprinus or carpio or killifish or eel or eels or anguilla or zander or sander or lucioperca or stizostedion or turbot or turbots or psetta or flatfish or flatfishes or plaice or pleuronectes or platessa or tilapia or tilapias or oreochromis or sarotherodon or common sole or dover sole or solea or zebrafish or zebrafishes or danio or rerio or seabass or dicentrarchus or labrax or morone or lamprey or lampreys or petromyzon or pumpkinseed or pumpkinseeds or lepomis or gibbosus or herring or clupea or harengus or amphibia or amphibian or amphibians or anura or salientia or frog or frogs or rana or toad or toads or bufo or xenopus or laevis or bombina or epidalea or calamita or salamander or salamanders or newt or newts or triturus or reptilia or reptile or reptiles or bearded dragon or pogona or vitticeps or iguana or iguanas or lizard or lizards or anguis fragilis or turtle or turtles or snakes or snake or aves or bird or birds or quail or quails or coturnix or bobwhite or colinus or virginianus or poultry or poultries or fowl or fowls or chicken or chickens or gallus or zebra finch or taeniopygia or guttata or canary or canaries or serinus or canaria or parakeet or parakeets or grasskeet or parrot or parrots or psittacine or psittacines or shelduck or tadorna or goose or geese or branta or leucopsis or woodlark or lullula or flycatcher or ficedula or hypoleuca or dove or doves or geopelia or cuneata or duck or ducks or greylag or graylag or anser or harrier or circus pygargus or red knot or great knot or calidris or canutus or godwit or limosa or lapponica or meleagris or gallopavo or jackdaw or corvus or monedula or ruff or philomachus or pugnax or lapwing or peewit or plover or vanellus or swan or cygnus or columbianus or bewickii or gull or chroicocephalus or ridibundus or albifrons or great tit or parus or aythya or fuligula or streptopelia or risoria or spoonbill or platalea or leucorodia or blackbird or turdus or merula or blue tit or cyanistes or pigeon or pigeons or columba or pintail or anas or starling or sturnus or owl or athene noctua or pochard or ferina or cockatiel or nymphicus or hollandicus or skylark or alauda or tern or sterna or teal or crecca or oystercatcher or haematopus or ostralegus or shrew or shrews or sorex or araneus or crocidura or russula or european mole or talpa or chiroptera or bat or bats or eptesicus or serotinus or myotis or dasycneme or daubentonii or pipistrelle or pipistrellus or cat or cats or felis or catus or feline or dog or dogs or canis or canine or canines or otter or otters or lutra or badger or badgers or meles or fitchew or fitch or foumart or foulmart or ferrets or ferret or polecat or polecats or mustela or putorius or weasel or weasels or fox or foxes or vulpes or common seal or phoca or vitulina or grey seal or halichoerus or horse or horses or equus or equine or equidae or donkey or donkeys or mule or mules or pig or pigs or swine or swines or hog or hogs or boar or boars or porcine or piglet or piglets or sus or scrofa or llama or llamas or lama or glama or deer or deers or cervus or elaphus or cow or cows or bos taurus or bos indicus or bovine or bull or bulls or cattle or bison or bisons or sheep or sheeps or ovis aries or ovine or lamb or lambs or mouflon or mouflons or goat or goats or capra or caprine or chamois or rupicapra or leporidae or lagomorpha or lagomorph or rabbit or rabbits or oryctolagus or cuniculus or laprine or hares or lepus or rodentia or rodent or rodents or murinae or mouse or mice or mus or musculus or murine or woodmouse or apodemus or rat or rats or rattus or norvegicus or guinea pig or guinea pigs or cavia or porcellus or hamster or hamsters or mesocricetus or cricetulus or cricetus or gerbil or gerbils or jird or jirds or meriones or unguiculatus or jerboa or jerboas or jaculus or chinchilla or chinchillas or beaver or beavers or castor fiber or castor canadensis or sciuridae or squirrel or squirrels or sciurus or chipmunk or chipmunks or marmot or marmots or marmota or suslik or susliks or spermophilus or cynomys or cottonrat or cottonrats or sigmodon or vole or voles or microtus or myodes or glareolus or primate or primates or prosimian or prosimians or lemur or lemurs or lemuridae or loris or bush baby or bush babies or bushbaby or bushbabies or galago or galagos or anthropoidea or anthropoids or simian or simians or monkey or monkeys or marmoset or marmosets or callithrix or cebuella or tamarin or tamarins or saguinus or leontopithecus or squirrel monkey or squirrel monkeys or saimiri or night monkey or night monkeys or owl monkey or owl monkeys or douroucoulis or aotus or spider monkey or spider monkeys or ateles or baboon or baboons or papio or rhesus monkey or macaque or macaca or mulatta or cynomolgus or fascicularis or green monkey or green monkeys or chlorocebus or vervet or vervets or pygerythrus or hominoidea or ape or apes or hylobatidae or gibbon or gibbons or siamang or siamangs or nomascus or symphalangus or hominidae or orangutan or orangutans or pongo or chimpanzee or chimpanzees or pan troglodytes or bonobo or bonobos or pan paniscus or gorilla or gorillas or troglodytes) \| 1 373 290 \| \| 19 \| #18 AND #17 \| 7 830 \| \| **20** \| **#19 and Meeting Abstract or Proceeding Paper or Editorial Material or Letter or Note (Exclude – Document Types)** \| **6 489** \| | |

| **Supplementary Table 1.** Description of the primary endpoints of the trials included in the network meta-analysis, presented per treatment group. AEs: Adverse Events; DFI: disease-free interval; DFS: Disease-Free Survival, EFS: Event-Free Survival, iDFS: invasive Disease-Free Survival, NS: Not Specified, RFS: Relapse-Free Survival | | | | | | | | | | | |
| --- | --- | --- | --- | --- | --- | --- | --- | --- | --- | --- | --- |
| **First author,**  **publication year** | **Study Name** | **Primary endpoint** | **Invasive in-breast recurrence** | **Regional recurrence** | **Distant recurrence** | **Death to breast cancer** | **Death to other causes** | **Invasive contralateral cancer** | **Ipsilateral DCIS** | **Contralateral DCIS** | **Other cancer** |
| **Strategies during first five postoperative years** | | | | | | | | | | | |
| Kaufmann, 2007 | ARNO-95 | DFS | ✓ | ✓ | ✓ | ✓ | ✓ | ✓ |  |  |  |
| Cuzick, 2010 | ATAC | DFS | ✓ | ✓ | ✓ | ✓ | ✓ | ✓ | ✓ | ✓ |  |
| Regan, 2011 | BIG 1-98 | DFS | ✓ | ✓ | ✓ | ✓ | ✓ | ✓ |  |  | ✓ |
| Dubsky, 2012 | ABCSG-8 | RFS | ✓ | ✓ | ✓ | ✓ |  |  |  |  |  |
| Boccardo, 2013 | ITA | DFS | ✓ | ✓ | ✓ |  |  |  | NS | NS |  |
| Aihara, 2014 | N-SAS BC03 | DFS | ✓ | ✓ | ✓ | ✓ | ✓ | ✓ | NS | NS | ✓ |
| Morden, 2017 | IES | DFS | ✓ | ✓ | ✓ | ✓ | ✓ | ✓ |  |  |  |
| Derks, 2017 | TEAM | DFS | ✓ | ✓ | ✓ | ✓ | ✓ | ✓ |  |  |  |
| De Placido, 2018 | FATA-GIM3 | DFS | ✓ | ✓ | ✓ | ✓ | ✓ | ✓ | ✓ | ✓ | ✓ |
| **Strategies incorporating ovarian function suppression** | | | | | | | | | | | |
| ABCTCG, 2007 | ABC OAS | OS |  |  |  | ✓ | ✓ |  |  |  |  |
| Hackshaw, 2009 | ZIPP | EFS | ✓ | ✓ | ✓ | ✓ | ✓ |  |  |  | ✓ |
| Tevaarwerk, 2014 | E-3193/INT-0142 | DFS | ✓ | ✓ | ✓ | ✓ | ✓ | ✓ | NS | NS |  |
| Gnant, 2014 | ABCSG-12 | DFS | ✓ | ✓ | ✓ | ✓ | ✓ | ✓ |  |  | ✓ |
| Yang, ASCO 2016 | NA | Estradiol, breast density, lipids |  |  |  |  |  |  |  |  |  |
| Perrone, 2019 | HOBOE | DFS | ✓ | ✓ | ✓ | ✓ | ✓ | ✓ | ✓ | ✓ | ✓ |
| Pagani, 2022 | SOFT/TEXT | DFS | ✓ | ✓ | ✓ | ✓ | ✓ | ✓ |  |  | ✓ |
| Francis, 2022 | SOFT | DFS | ✓ | ✓ | ✓ | ✓ | ✓ | ✓ |  |  | ✓ |
| Kim, 2023 | ASTRRA | DFS | ✓ | ✓ | ✓ | ✓ | ✓ | ✓ |  |  | ✓ |
| **Extended endocrine therapy** | | | | | | | | | | | |
| Fisher, 2001 | NSABP-B14 | DFS | ✓ | ✓ | ✓ | ✓ | ✓ | ✓ |  |  | ✓ |
| Stewart, 2001 | Scottish | Relapse |  |  | ✓ | ✓ | ✓ | ✓ |  |  |  |
| Jakesz, 2007 | ABCSG 6a | RFS | ✓ | ✓ | ✓ |  |  | ✓ |  |  |  |
| Mamounas, 2008 | NSABP B-33 | DFS | ✓ | ✓ | ✓ | ✓ | ✓ | ✓ | ✓ | ✓ | ✓ |
| Ingle, 2008 | MA.17 | DFS | ✓ | ✓ | ✓ |  |  | ✓ | ✓ | ✓ |  |
| Davies, 2013 | ATLAS | RFS | ✓ | ✓ | ✓ |  |  | ✓ | NS | NS |  |
| Gray, 2013 | aTTom | DFI | ✓ | ✓ | ✓ |  |  |  |  |  |  |
| Blok, 2018 | IDEAL | DFS | ✓ | ✓ | ✓ | ✓ | ✓ | ✓ | ✓ | ✓ |  |
| Del Mastro, 2021 | GIM4 | DFS | ✓ | ✓ | ✓ | ✓ | ✓ | ✓ |  |  | ✓ |
| Gnant, 2021 | ABCSG-16 | DFS | ✓ | ✓ | ✓ | ✓ | ✓ | ✓ |  |  | ✓ |
| Mamounas, 2023 | NSABP B-42 | DFS | ✓ | ✓ | ✓ | ✓ | ✓ | ✓ |  |  | ✓ |
| Iwase, 2023 | AERAS | DFS | ✓ | ✓ | ✓ | ✓ | ✓ | ✓ |  |  | ✓ |
| Tjan-Heijnen, 2023 | DATA | DFS | ✓ | ✓ | ✓ | ✓ | ✓ | ✓ | ✓ | ✓ | ✓ |
| **Addition of targeted agents** | | | | | | | | | | | |
| Mayer, 2021 | PALLAS | iDFS | ✓ | ✓ | ✓ | ✓ | ✓ | ✓ |  |  | ✓ |
| Loibl, 2021 | Penelope-B | iDFS | ✓ | ✓ | ✓ | ✓ | ✓ | ✓ |  |  | ✓ |
| Bachelot, 2022 | UNIRAD | DFS | ✓ | ✓ | ✓ | ✓ | ✓ | ✓ |  |  | ✓ |
| Chavez, 2024 | SWOGS1207 | iDFS | ✓ | ✓ | ✓ | ✓ | ✓ | ✓ |  |  | ✓ |
| Hortobagyi, 2023 | NATALEE | iDFS | ✓ | ✓ | ✓ | ✓ | ✓ | ✓ |  |  | ✓ |
| Rastogi, 2024 | monarchE | iDFS | ✓ | ✓ | ✓ | ✓ | ✓ | ✓ |  |  | ✓ |

| **Supplementary Table 2.** Randomized trials that were excluded from the meta-analysis | | | | |
| --- | --- | --- | --- | --- |
| **First author, publication year** | **Study Name** | **Identifier** | **Comparison** | **Reason for exclusion** |
| Goss, 2013 | MA.27 | NCT00066573 | Exemestane vs Anastrozole | Comparison between two aromatase inhibitors |
| Smith, 2017 | FACE | NCT00248170 | Letrozole vs Anastrozole | Comparison between two aromatase inhibitors |
| Jerusalem, 2021 | SOLE | NCT01864746 | Continuous vs intermittent treatment | Only study to evaluate intermittent adjuvant endocrine treatment |
| Ruiz-Borrego, 2019 | GEICAM/ 2006-10 | NCT00543127 | Anastrozole with or without Fulvestrant | Only study to evaluate adjuvant fulvestrant |
| Li, 2019 | NA | NCT01352091 | 5 years TAM vs switch (TAM and AI+OFS) | Only study to evaluate switch strategy in premenopausal patients |
| Goss, 2016 | MA.17R | NCT00754845 | Additional 5 years AI to TAM and then AI | Only study to evaluate 15 versus 10 years of endocrine therapy (for most, but not all included patients) |

| **Supplementary Table 3.** Adverse events of endocrine treatment strategies for premenopausal patients during the first five postoperative years. Abbreviations: OFS: ovarian function suppression; Tam: tamoxifen; AI: aromatase inhibitor; DVT: deep venous thrombosis; CI: confidence interval | | | | | | |
| --- | --- | --- | --- | --- | --- | --- |
|  | **OFS + Tam vs Tam** | | **OFS + AI vs Tam** | | **OFS + AI vs OFS + Tam** | |
| **Adverse event** | **Pooled rates** | **Odds ratio (95% CI)** | **Pooled rates** | **Odds ratio (95% CI)** | **Pooled rates** | **Odds ratio (95% CI)** |
| DVT | 1.8% vs 2.2% | 1.05 (0.70-1.60) | 0.8% vs 2.2% | 0.52 (0.34 – 0.81) | 0.8% vs 1.8% | 0.50 (0.33 – 0.74) |
| Osteoporosis | 27.9% vs 13.7% | 2.42 (2.08 – 2.81) | 42.2% vs 13.7% | 4.58 (3.94 – 5.32) | 42.2% vs 27.9% | 1.89 (1.69 – 2.11) |
| Fracture | 5.3% vs 4.7% | 1.15 (0.90 – 1.48) | 6.0% vs 4.7% | 1.50 (1.17 – 1.92) | 6.0% vs 5.3% | 1.30 (1.06 – 1.59) |
| Arthralgia | 6.6% vs 0.9% | 2.76 (0.82 – 9.25) | 21.0% vs 0.9% | 7.65 (2.21 – 26.47) | 21.0% vs 6.6% | 2.77 (2.09 – 3.68) |
| Hot Flushes | 88.1% vs 69.4% | 3.52 (2.94 – 4.22) | 92.4% vs 69.4% | 2.98 (2.49 – 3.57) | 92.4% vs 88.1% | 0.85 (0.70 – 1.02) |
| Hypertension | 22.0% vs 15.4% | 1.41 (1.22 – 1.63) | 24.3% vs 15.4% | 1.46 (1.26 – 1.69) | 24.3% vs 22.0% | 1.04 (0.92 – 1.17) |

| **Supplementary Table 4.** Adverse events of endocrine treatment strategies for postmenopausal patients during the first five postoperative years. Abbreviations: Tam: tamoxifen; AI: aromatase inhibitor; DVT: deep venous thrombosis; CI: confidence interval | | | | | | |
| --- | --- | --- | --- | --- | --- | --- |
|  | **Switch vs Tam** | | **AI vs Tam** | | **AI vs Switch** | |
| **Adverse event** | **Pooled rates** | **Odds ratio (95% CI)** | **Pooled rates** | **Odds ratio (95% CI)** | **Pooled rates** | **Odds ratio (95% CI)** |
| DVT | 1.8% vs 3.9% | 0.80 (0.53 – 1.21) | 1.7% vs 3.9% | 0.53 (0.38 – 0.73) | 1.7% vs 1.8% | 0.66 (0.45 – 0.97) |
| Osteoporosis | 9.0% vs 5.4% | 1.33 (0.94 – 1.88) | 11.2% vs 5.4% | 1.95 (1.42 – 2.68) | 11.2% vs 9.0% | 1.47 (1.09 – 1.99) |
| Fracture | 8.0% vs 5.2% | 1.37 (1.06 – 1.77) | 8.4% vs 5.2% | 1.48 (1.15 – 1.90) | 8.4% vs 8.0% | 1.08 (0.83 – 1.40) |
| Arthralgia | 22.2% vs 20.8% | 1.63 (1.27 – 2.10) | 28.9% vs 20.8% | 1.59 (1.23 – 2.05) | 28.9% vs 22.2% | 0.97 (0.72 – 1.31) |
| Hot Flushes | 46.8% vs 52.6% | 1.01 (0.92 – 1.10) | 31.0% vs 52.6% | 0.81 (0.74 – 0.89) | 31.0% vs 46.8% | 0.80 (0.73 – 0.89) |

| **Supplementary Table 5.** Adverse events of extended endocrine treatment strategies following five years of tamoxifen. Abbreviations: Tam: tamoxifen; AI: aromatase inhibitor; DVT: deep venous thrombosis; CI: confidence interval; NA: not available | | | | |
| --- | --- | --- | --- | --- |
|  | **5 years Tam vs no extension** | | **5 years AI vs no extension** | |
| **Adverse event** | **Pooled rates** | **Odds ratio (95% CI)** | **Pooled rates** | **Odds ratio (95% CI)** |
| DVT | 0.6% vs 0.3% | 1.95 (1.14 – 3.32) | NA | NA |
| Osteoporosis | NA | NA | 8.1% vs 6.0% | 1.38 (1.11 – 1.72) |
| Fracture | 1.0% vs 2.1% | 1.19 (0.24 – 5.87) | 4.9% vs 2.1% | 1.20 (0.95 – 1.42) |
| Arthralgia | NA | NA | 25.3% vs 20.3% | 1.30 (1.23 – 1.48) |
| Hot Flushes | NA | NA | 57.8% vs 48.9% | 1.18 (1.06 – 1.32) |

| **Supplementary Table 6.** Adverse events of extended endocrine treatment strategies following five years of aromatase inhibitor or switch strategy. Abbreviations: AI: aromatase inhibitor; DVT: deep venous thrombosis; CI: confidence interval | | | | |
| --- | --- | --- | --- | --- |
|  | **2-3 years AI vs no extension** | | **5 years AI vs no extension** | |
| **Adverse event** | **Pooled rates** | **Odds ratio (95% CI)** | **Pooled rates** | **Odds ratio (95% CI)** |
| DVT | 0.5% vs 0.1% | 4.55 (0.35 - 59.65) | NA | NA |
| Osteoporosis | 11.9% vs 20.6% | 1.28 (0.75 - 2.18) | 28.6% vs 20.6% | 1.59 (0.84 - 3.00) |
| Fracture | 4.5% vs 5.3% | 1.14 (0.77 - 1.68) | 6.8% vs 5.3% | 1.50 (1.00 - 2.25) |
| Arthralgia | 34.6% vs 36.6% | 1.27 (1.12 - 1.44) | 25.1% vs 36.6% | 1.44 (1.21 - 1.71) |
| Hot Flushes | 11.8% vs 11.2% | 1.11 (0.88 - 1.39) | 13.5% vs 11.2% | 1.42 (1.12 - 1.81) |

| **Supplementary Table 7.** Number of patients needed to treat (NNT) to avoid one breast cancer relapse in the various escalation endocrine treatment strategies. Abbreviations: OFS: ovarian function suppression; AI: aromatase inhibitor | |
| --- | --- |
| **Strategy** | **NNT** |
|  |  |
| OFS + tamoxifen versus tamoxifen (at 8 years) | 25 |
| OFS + AI versus tamoxifen (at 8 years) | 14 |
| OFS + AI versus OFS + tamoxifen (at 8 years) | 29 |
| 10 years tamoxifen versus 5 years tamoxifen (at 13 years) | 35 |
| 5 years AI + 5 years tamoxifen versus 5 years tamoxifen (at 13 years) | 13 |
| 5 years AI + 5 years tamoxifen versus 10 years tamoxifen (at 13 years) | 21 |
| 8 years AI versus 5 years AI (at 13 years) | 28 |
| 10 years AI versus 5 years AI (at 13 years) | 24 |
| Addition of CDK4/6 inhibitor (at 4 years) | 28 |

**Supplementary Figure 1.** Graphic description of the geographical distribution of the countries involved in the included trials, divided into groups on the number of trials.

**Supplementary Figure 2.** Risk of Bias evaluation based on the RoB2 tool

**Supplementary Figure 3.** Consistency of the network estimates for the comparisons between different strategies. Abbreviations: ET: endocrine therapy; OFS: ovarian function suppression; AI: aromatase inhibitor; HR: hazard ratio; CI: confidence interval

**
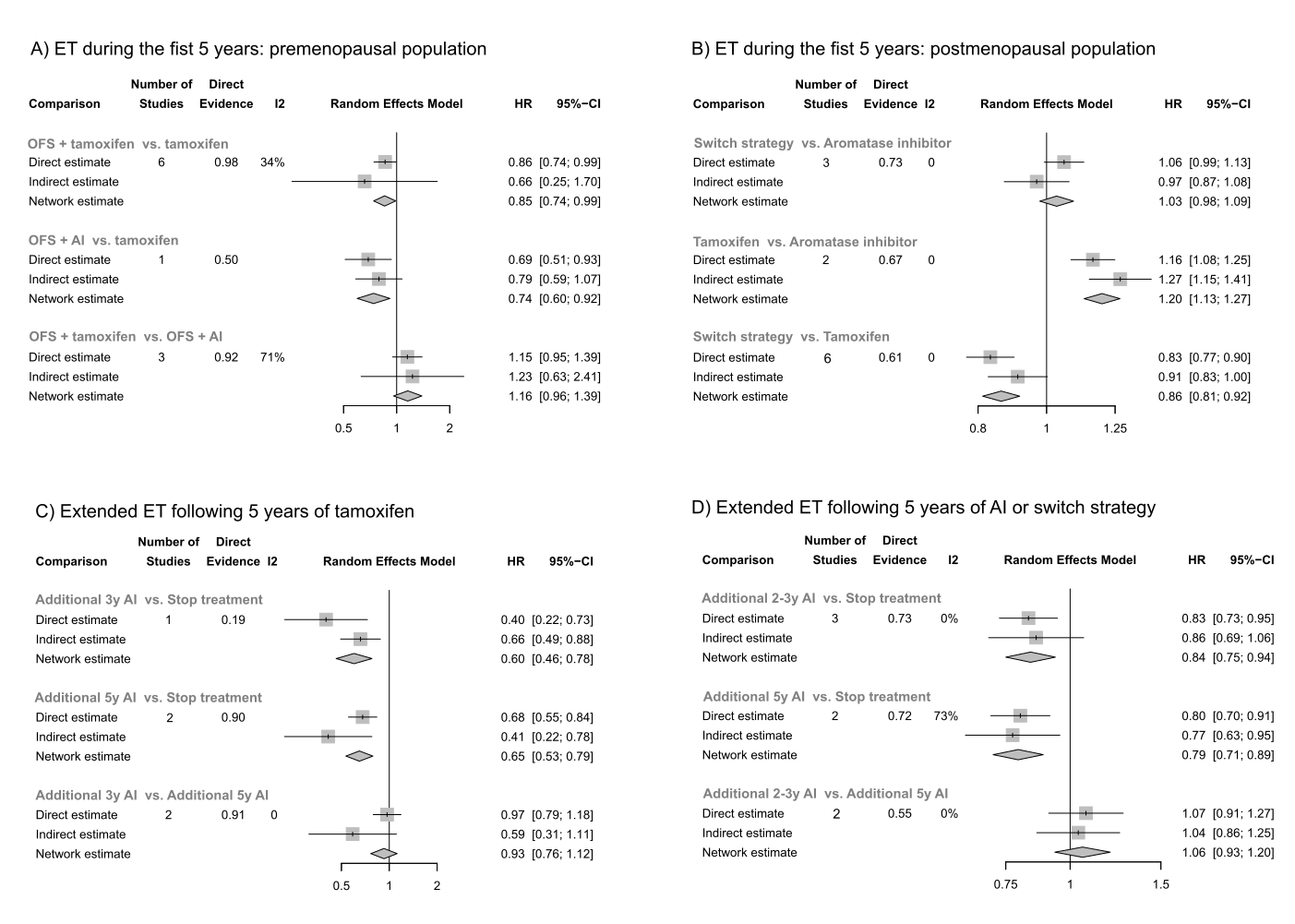
**

**Supplementary Figure 4.** Comparison of treatment strategies during the first five postoperative years. Trial-level network meta-analysis for the overall survival endpoint in A) premenopausal and B) postmenopausal patients. Abbreviations: OFS: ovarian function suppression; AI: aromatase inhibitors; HR: hazard ratio; CI: confidence interval.


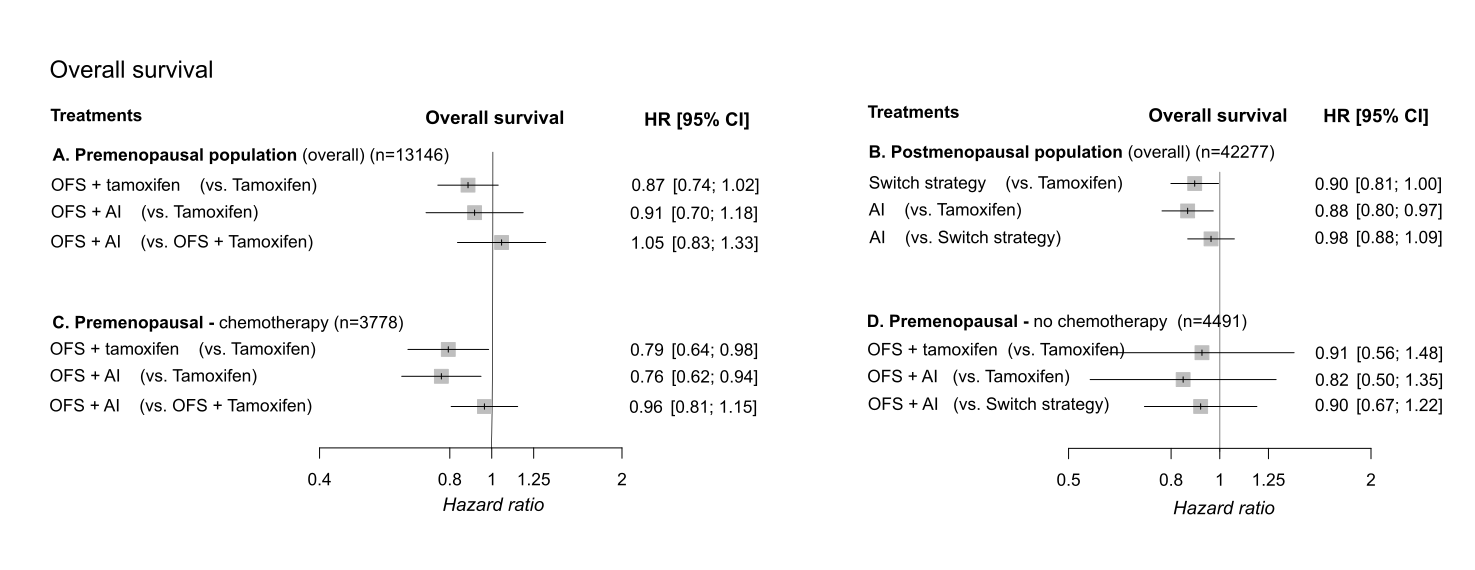


**Supplementary Figure 5.** Comparison of extended treatment with aromatase inhibitors of any duration compared with five years of aromatase inhibitors with or without tamoxifen. Abbreviations: PR: progesterone receptor; HR: hazard ratio; CI: confidence interval.

**
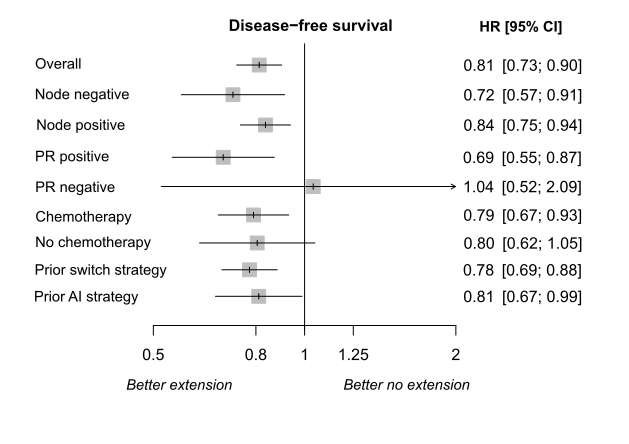
A)**

**
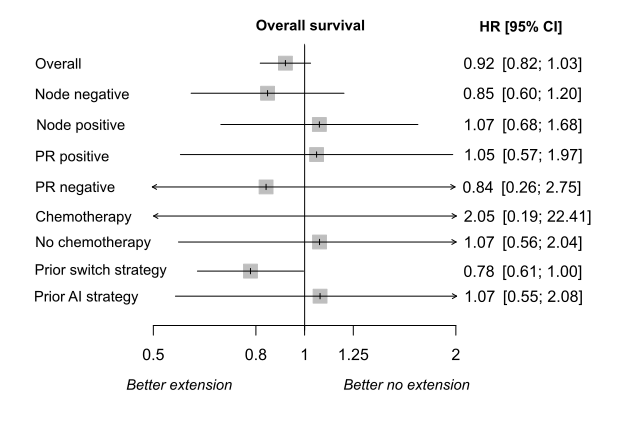
 B)**

**Supplementary Figure 6.** Comparison of treatment strategies following five years of aromatase inhibitor or switch strategy. Trial-level network meta-analysis for the overall survival endpoint in the overall population and subgroups of interest. Abbreviations: OFS: ovarian function suppression; AI: aromatase inhibitors; TAM: tamoxifen; PR: progesterone receptor; N: nodal status; HR: hazard ratio; CI: confidence interval.


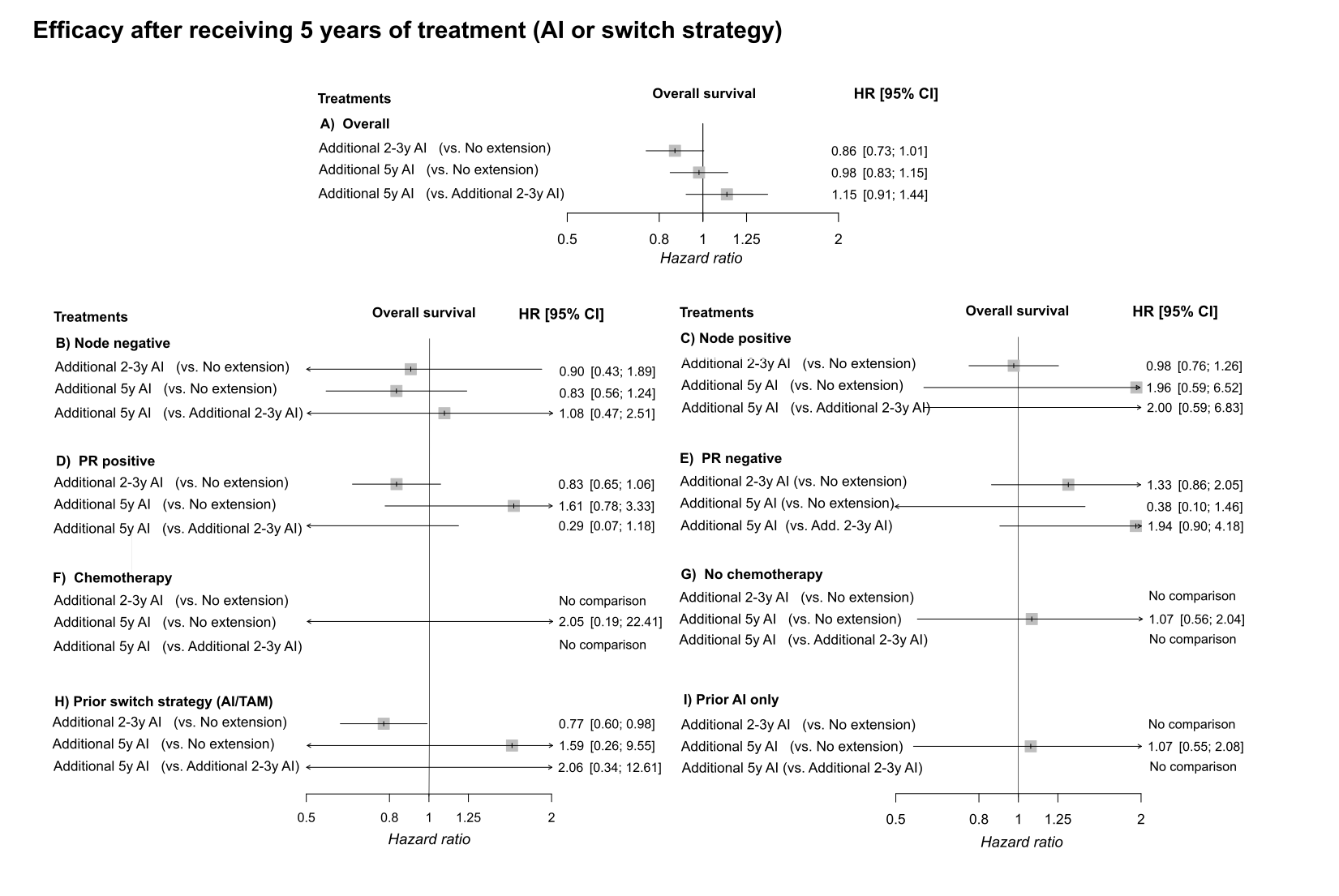


**Supplementary Figure 7.** Comparison of addition of molecularly targeted agents. A) trial-level network meta-analysis. Comparisons in terms of disease-free survival in entire population (A1) and according to menopausal status (A2), nodal status (A3) and timing of chemotherapy (A4). B) Kaplan-Meier curves for disease-free survival, generated with extracted individual patient data, comparing two treatment strategies of no endocrine therapy alone or with addition of cyclin dependent kinases 4/6 inhibitors. Abbreviations: DFS: disease-free survival; ET: endocrine treatment; HR: hazard ratio; CI: confidence interval; CDK4/6i: cyclin dependent kinases 4/6 inhibitor; mTORi: mammalian target of rapamycin inhibitor

**
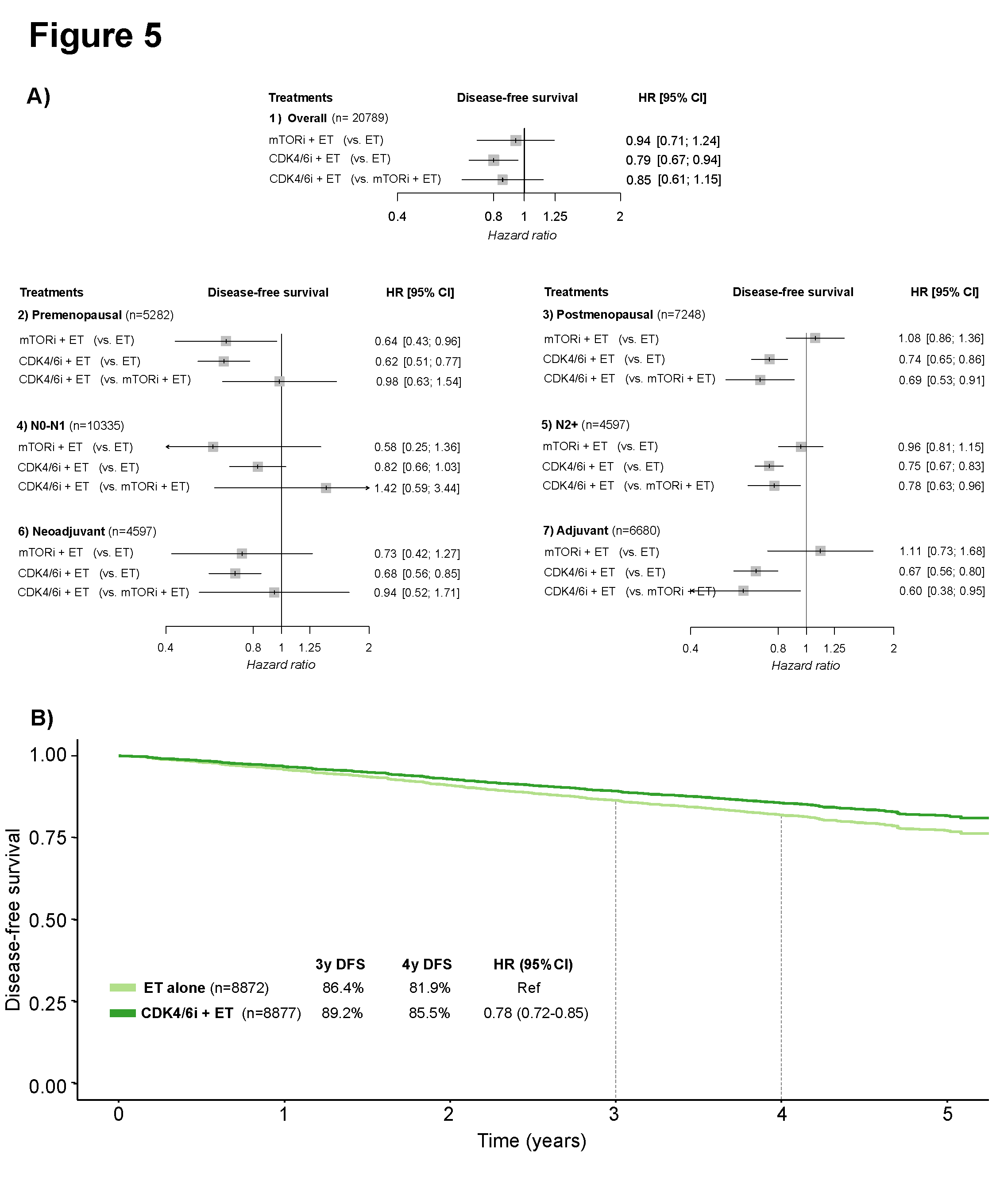
**

**Supplementary Figure 8.** Comparison of addition of molecularly targeted therapy to endocrine therapy. Trial-level network meta-analysis for the overall survival endpoint. Abbreviations: mTORi: inhibitors of the mammalian target of rapamycin; CDK4/6i: inhibitors of cyclin dependent kinases 4 and 6; ET: endocrine therapy; HR: hazard ratio; CI: confidence interval.


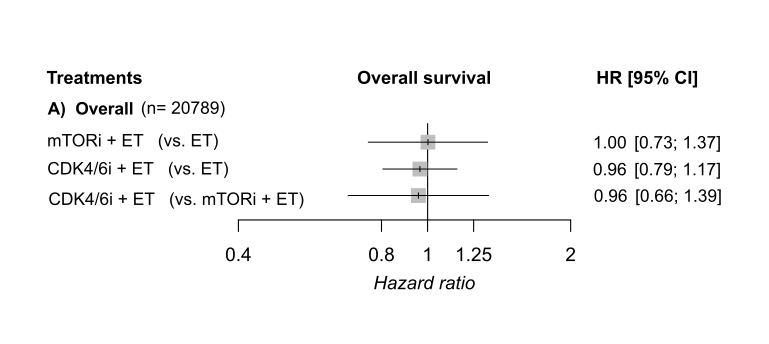

Supplement: Supplementary Data [file mmc1.docx]
